# Leveraging local species data, a global database, and an occupancy model to explore bee–plant interactions

**DOI:** 10.1002/eap.70221

**Published:** 2026-03-24

**Authors:** Michelle J. Lee, Graziella V. DiRenzo, Chengyi Diao, Katja C. Seltmann

**Affiliations:** ^1^ Ecology, Evolution and Marine Biology University of California Santa Barbara California USA; ^2^ School of Environment, Society & Sustainability, University of Utah Salt Lake City Utah USA; ^3^ U.S. Geological Survey, Massachusetts Cooperative Fish and Wildlife Research Unit University of Massachusetts Amherst Amherst Massachusetts USA; ^4^ Cheadle Center for Biodiversity and Ecological Restoration, University of California Santa Barbara Santa Barbara California USA

**Keywords:** bee–plant interactions, California, community science, imperfect detection, occupancy modeling, opportunistic data, pollinators, presence‐only, Santa Cruz Island

## Abstract

Global declines in bee populations are threatening the ecosystem services they provide, including pollination. Many bee–plant interactions are understudied, producing an incomplete understanding of resulting ecosystem‐level vulnerabilities. The last decade has generated a wealth of opportunistic data originating from natural history collection records, published ecological datasets, and citizen/community science initiatives in online databases such as Global Biotic Interactions (GloBI). Here, we explore hypotheses related to bee–plant interactions and detection processes using the GloBI database, curated checklists of bee and flowering plant species, and an occupancy model. We hypothesized that larger, social bees would visit a larger number of plant species, while smaller, solitary bees would visit fewer. We also predicted that flowers with open, bowl‐like shapes would attract a greater diversity of bee visitors compared to closed shapes. Further, we hypothesized that both floral and bee traits, such as bright colors and conspicuous patterns, would increase detectability, and that different data collection methods would vary in their ability to capture bee–plant interactions. Lastly, we hypothesized that the interaction network generated by the output of the occupancy model, which accounted for imperfect bee–plant detection, would yield more interactions, thereby increasing measures of evenness and decreasing nestedness and specialization, as compared to the network generated from recorded interaction data. We found that smaller bees exhibited higher probabilities of plant interactions than larger bees, but we did not find evidence that bee sociality influenced the probability of interacting with plants. We found that blue flowers and closed (not‐bowl‐shaped) flowers had higher probabilities of bee‐plant interaction than other flower colors or bowl‐shaped flowers, respectively. We also found that larger bee size, blue flowers, bowl shapes, and community science sources were associated with higher detection probabilities of bee–plant interactions. Lastly, the interaction network generated by the occupancy model output showed higher levels of evenness, nestedness, and connectance than the network generated by the GloBI data. Our study is among the first to utilize occupancy modeling to directly model species' interactions, leverage aggregated, open‐source databases and expert checklists, and highlight the influence of detection and collection biases on our understanding of ecological interactions.

## INTRODUCTION

Declines in the abundance and diversity of bees have been reported globally and regionally, with resulting declines in bee interactions and pollination services (Potts et al., 2010; Zattara & Aizen, 2021). Currently, our ability to evaluate the decline of the ca. 20,000 bee species worldwide, their interactions, and thus community structure relies heavily on opportunistic occurrence data found in museum record databases, natural history collections, and community science initiatives (Arce et al., 2023; Soroye et al., 2020; Souther et al., 2024; Zattara & Aizen, 2021), which are often influenced by observer and sampling biases. Over two billion records documenting the global occurrence of organisms are available on platforms such as Global Biodiversity Information Facility (GBIF), Integrated Digitized Biocollections (iDigBio), iNaturalist, and others. These records span all taxonomic kingdoms, and most of these records are obtained from citizen/community science initiatives (referred to as community science moving forward), atlas data, and the digitization of natural history collection specimen records. Typically, these data are not collected according to a specific study or design, making them a type of “opportunistic” data. The availability of opportunistic data is only increasing with the rise of online platforms, and they continually provide new evidence of species occurrences across space and time (Heberling et al., 2021). When using opportunistic data, several biases can emerge, including: (1) taxonomic bias (e.g., some species may be sampled more frequently than others because they are easier to sample, better known, targeted for inference, or receive prioritized funding); (2) detection bias (e.g., species detectability varies due to observer experience, survey frequency, or inherent differences in species conspicuousness); and (3) spatial bias (e.g., some locations are more easily accessed). Although these biases exist, several studies have successfully used opportunistic data to examine species distributions across space and time (e.g., Kéry et al., 2010), including population declines (e.g., Guzman et al., 2021), and species range shifts and contractions (e.g., Tingley & Beissinger, 2009). These studies have improved our understanding of how species distributions have been impacted by threats and stressors, but they typically lack the exploration of how species interactions change.

In our understanding of the ecological dynamics of bee–plant interactions, two key questions emerge: (1) which bee characteristics are associated with a species' degree of plant interaction generalization, and (2) which bee, floral, and observer traits influence the detectability of a bee–plant interaction? Previous research has provided valuable insights into these topics. For example, bee traits, such as body size and sociality, are particularly important predictors of diet breadth. Larger bees, with greater flight capacity and resource demands, often have generalized diets that include a wide range of plant species (Cullen et al., 2021; Greenleaf et al., 2007). Likewise, social bees, such as bumble bees and honey bees, tend to have more generalized diets and often forage in large groups across diverse floral resources within their range (Kaluza et al., 2018; Wood et al., 2018). Additionally, floral traits, such as shape and color, influence bee foraging behavior and floral preferences. For example, open flower shapes (e.g., bowl‐like shapes) may attract more generalist bee species than closed flower shapes (e.g., not‐bowl‐like shapes; Wang et al., 2024). Pollinators may be attracted to floral traits that signal higher resource rewards, such as vibrant colors (Dafni et al., 1990; Waser & Price, 1981). Bees have a reported color vision of 300–630 nm (Kevan, 1972), and blue and purple colors are associated with high attraction and visitation (Acharya et al., 2021; Reverté et al., 2016). While white and yellow colors are highly attractive colors for broad arrays of pollinators, they are less associated with bee attraction and visitation (Acharya et al., 2021; Wang et al., 2024).

In addition to ecological factors shaping bee–plant interaction networks, detection processes are also influenced by species‐specific traits and professional and nonprofessional observer‐related factors. Bee traits, such as size and stripiness (if the bee has stripes or not), may increase detectability because larger or more distinctively patterned bees are easier for observers to notice. However, detectability is also shaped by the method of data collection. For example, professional researchers, who often have extensive training, may target specific taxa according to their own research objectives, potentially introducing taxonomic biases (Adamo et al., 2021). In contrast, community science initiatives, while often more spatially extensive, may be biased toward charismatic or easily identifiable species (Di Cecco et al., 2021; Goldstein et al., 2024; Stoudt et al., 2022). Similarly, natural history collections tend to overrepresent rare or visually striking taxa, which can result in underrepresentation of common or less conspicuous species. As a result, the interaction networks documented by professional researchers, community science initiatives, and natural history collections may each capture only a subset of the full range of interactions, potentially skewing ecological community structure estimates.

A powerful tool for disentangling these ecological and sampling processes is occupancy modeling. Occupancy models retain the identity of individual species while accounting for variable and imperfect detection, which is not always the case for traditional methods for quantifying biodiversity (Iknayan et al., 2014; Kéry & Royle, 2016). Because these models require standardized data collection and adherence to specific assumptions, deviations can lead to biased parameter estimates and flawed ecological inferences (Kéry & Royle, 2016). Nevertheless, researchers have devised approaches to adapt occupancy models for non‐standardized, opportunistic data (Robinson et al., 2020; Shirey et al., 2023).

Accounting for imperfect detection can improve estimates of species interaction networks and community structure. Incorporating additional data sources and correcting for incomplete or biased observations may improve the detection of rare interactions and yield more comprehensive estimates of species interactions. This approach is expected to produce networks with increased evenness, reduced nestedness (i.e., less hierarchical structuring where rare species interact only with common ones), and reduced specialization. Because metrics like nestedness and specialization are often overestimated due to species rarity and sampling biases (Rivera‐Hutinel et al., 2012), addressing these limitations will enable researchers to develop more accurate representations of community structure, which are critical for evaluating ecosystem resilience and stability.

Here, we explored hypotheses related to bee and floral characteristics that may drive both ecological processes (i.e., what bee and plant species characteristics are associated with their degree of interactions?) and detection processes (i.e., what bee, plant, and dataset characteristics affect detectability?). We further examined how accounting for detection biases alters the inferred structure of interaction networks. Specifically, we hypothesized that larger bees with greater flight capacity, as well as social bees that forage in groups, would visit more plant species, while smaller, solitary bees would visit fewer plant species. We also expected floral traits, such as open flower shapes, to support a broader diversity of bee visitors due to easier resource access, and that brightly colored flowers may increase detectability by both bees and observers. Regarding detection processes, we predicted that larger and more conspicuous bees (e.g., those with stripes or bright colors) would be more easily observed. Further, data collection methods may introduce additional biases, with professional collectors potentially targeting specific taxa and community science data overrepresenting some species. Lastly, we hypothesized that by explicitly accounting for detection biases, the bipartite network generated by the occupancy model output would exhibit greater evenness and reduced nestedness than a bipartite network generated from observed interaction data. Specifically, incorporating occupancy‐based estimates will uncover under‐sampled bee–plant interactions that remain obscured in raw or normalized bee–plant interaction records.

We approached these questions using the following framework: (1) we compiled a comprehensive dataset of species checklists, trait data, and interaction records from an online aggregated interaction database (Global Biotic Interactions, GloBI, Poelen et al., 2014) to characterize the full scope of potential bee–plant interactions; (2) we implemented an occupancy model to account for imperfect species detection and improve interaction estimates; (3) we compared occupancy model parameter estimates to determine which covariates explain bee–plant interactions and their detectability; and (4) we calculated network metrics for two bipartite networks (i.e., one from the occupancy model output, and one from the GloBI data) to understand the differences between the bee–plant interactions predicted from each approach. Through this analysis, we aimed to disentangle ecological processes from detection biases to better understand the drivers of bee–plant interactions.

## METHODS

### GloBI database and species checklists

GloBI is an online integrated information system for indexing and sharing species interaction data (Poelen et al., 2014). It aggregates datasets that include interactions derived from natural history collections and community science projects with records from one‐time observers via online occurrence record databases (e.g., iNaturalist). GloBI also includes data from research studies, such as meta‐analyses and ecological studies, worldwide. The data sources vary in terms of objectives and study design, and the GloBI database indexes these records uniformly. While GloBI includes the source citations (i.e., project that includes a sampling event or observation event) of where the interaction was published with each interaction record and geographic information if it is provided by the source, the user/practitioner must develop methods for inferring the absence or non‐detection of species interactions. Here, we proposed the use of species checklists to infer species interaction non‐detections (e.g., Kéry et al., 2010).

Species checklists are authoritative lists of species that occur in a geographical area. Checklists are often generated by taxonomists and collectors specializing in a particular taxonomic group and provide an important standard for decision‐making in biodiversity conservation and land management (Johnson et al., 2020; Reyserhove et al., 2020). Checklists are usable to retroactively assign species non‐detections by comparing what was observed to a list of species that are expected to occur at a site (Kéry et al., 2010). We can then infer species and interaction non‐detections.

### Data normalization and formatting

To explore the hypotheses related to bee and plant interactions and detection, we used plant and bee species checklists, bee trait data (i.e., body size, stripiness, sociality), plant trait data (i.e., flower color and shape), and species interaction data from GloBI. We took the following steps to prepare the GloBI data:

1. *Identifying bee–plant communities*: We identified Santa Cruz Island, California, as the focal study area to explore the hypotheses based on the accessibility of authoritative species checklists for both bees and plants. Bees and plants have been extensively monitored and studied on the island for more than 10 years. Bee taxonomic specialists Robbin W. Thorp and John S. Ascher provided the first extensive checklist of the island in 2007 (Thorp, 2007), with many additions to the list since (Seltmann et al., 2022). The plant checklist (Hasenstab‐Lehman et al., 2022) is based on vouchered specimens in natural history collections and continues to be actively updated.
  We used records of bee–plant interactions from the GloBI database, retaining only species‐level interactions in which both taxa appeared on our Santa Cruz Island checklists. We did not apply geographic filters because these constraints greatly reduced the number of records, and species pairs documented elsewhere were considered potentially observable on Santa Cruz Island.
2. *Normalized species checklists*: The bee checklist for Santa Cruz Island in June 2022 had a total of 142 species. We excluded any species in our checklists not identified to species level—that is, genus is identified but listed as “sp.” This removed a total of four bee taxa (*Calliopsis* sp., *Sphecodes* sp., *Stelis* sp., *Nomada* sp.) that likely represent multiple species. We also excluded *Apis mellifera* from the bee checklist because *A. mellifera* was extirpated from Santa Cruz Island in ca. 2004. Lastly, we removed four species (*Colletes kincaidii*, *Bombus occidentalis*, *Halictus harmonius*, and *Eucera lunata*) because they are thought to be misidentifications. Our final list of bee species included 133 species.
  The original plant checklist consisted of 562 species. We excluded all nonflowering plant species, and we excluded flowering plant genera that had no recorded bee–plant interaction in our GloBI interaction list (including plants that had an interaction with *A. mellifera*). This brought our total plant list to 302 species.
3. *Compiling bee and plant trait data*: We obtained bee size, sociality, and coloration data from original descriptions and images on Discover Life (Ascher & Pickering, 2020), using “The Bees in Your Backyard” (Wilson & Carril, 2015), Bee Library (Seltmann et al., 2021), BugGuide, GBIF, and iNaturalist (Lee et al., 2025). We checked for collinearity among traits before conducting analyses (Pearson's correlation coefficient, all *r* < 0.7; Dormann et al., 2013) and retained all covariates.
  Bee sociality was divided into solitary and social categories, where solitary includes communal, aggregate, and parasitic lifestyles. Social bees include those bees that are eusocial, and those that are primitively or facultatively eusocial (Danforth et al., 2019; Ostwald et al., 2024). Social status was largely inferred at the genus level, except within the Halictidae family where species‐level was investigated due to the high amount of variation, especially within the *Lasioglossum* genus. In cases where sociality of the species within the *Lasioglossum* genus was not known, we assumed solitary except for those in the subgenus *Hemihalictus* because the majority of the non‐*Hemihalictus Lasioglossum* bees are considered solitary (Brady et al., 2006; Soucy, 2002).
  We collected data related to floral color and shape using Calflora and Jepson eFlora following the designations of Bartomeus, 2013, Bosch et al., 1997, and Olesen et al., 2007. We classified flowers as bowl‐shaped (i.e., wide, open, concave petals forming a bowl‐like structure) or not‐bowl‐shaped (e.g., narrow, closed, convex petals forming a not‐bowl‐like structure) because these broad descriptors were less likely to mischaracterize the category (Appendix S1: Table S1). We classified flower color as either yellow, white, blue/purple, or other colored flowers as these colors are common bee and general insect pollinator attractants. We included purple flowers into the blue flower grouping because these colors were not easily distinguished during classification. Other colored flowers include pink, green, orange, and red. More details related to our trait search can be found in Appendix S1: Methods.
4. *GloBI data normalization*: We downloaded all unique interactions with bee species from GloBI (Poelen et al., 2014) on 18 January 2024, which consisted of 840,315 observations (Seltmann et al., 2025). As of January 2024, some occurrence data in GloBI included duplicates from recent reviews of published interactions. We checked for these potential duplicates using unique code combinations associated with museum specimen collection and catalog numbers. After removing these duplicates, 656,525 bee‐related interactions remained.
  4.1. *Aligning plant species names and filtering to only plant species in the checklist*: Interaction data from GloBI are directional. Thus, we first standardised the columns where bee and plant species names appeared, given that they could be in either the target or source columns of the GloBI data.
    After the species names were standardized, we ran the TNRS package in R (Maitner & Boyle, 2021; R Core Team, 2023) on the plant column to obtain the accepted name per plant species according to World Flora Online (World Flora Online, 2023). We removed any observations that did not include both a bee and plant identified to species. We then limited the dataset to those with plant species in our plant checklist. These steps brought the total number of recorded interactions down to 380,597 entries.
  4.2. *Aligning bee species names and filtering to only bee species in the checklist*: To ensure that we obtained all GloBI entries for the bees in our checklists, we created a bee synonym list from Discover Life bee species guide and world checklist (Hymenoptera: Apoidea: Anthophila; Ascher & Pickering, 2020) accessed via Zenodo (Seltmann & Poelen, 2021). In addition, we used data from Dorey et al. (2023) accessed via Zenodo (Poelen & Seltmann, 2024) and the Big‐Bee Network name alignment tool (Seltmann, 2025). The Big‐Bee Network name alignment tool uses *nomer* software (Salim & Poelen, 2025), which is the same tool used by GloBI. Nomer provides a flexible mechanism for creating custom catalog lists to align taxon names. We compared this list to all bee names in the GloBI dataset. The results from the name alignment are available in our data release (Lee et al., 2025). After we aligned all bee and plant species names in the GloBI dataset, we filtered the data to only include bee and plant species in our checklist, leaving 10,836 entries.
  4.3. *Normalizing source citation information*: Next, we normalized the source citation information. We used a combination of the source institution code and source citation to generate unique source citations, given that multiple source institution codes can be buried within source citation. We named this new variable “resolved source citation.” Therefore, although our filtered GloBI dataset (subsetted to only bee and plant interactions occurring in our checklist) included 33 source citations, our resolved source citation included 50 sources (Appendix S1: Table S2).
    We categorized each source as either aggregated data (i.e., data are collected from other sources; e.g., web scraping), collection specimen (e.g., museum), literature (i.e., primary published literature), or observation (i.e., community science initiatives; e.g., iNaturalist).
    Lastly, we subsetted the GloBI dataset to only sources with >99 unique bee–plant interactions documented to ensure that each source aimed to record all possible bee–plant observations rather than targeted sampling. This produced a total of six source citations that met this criterion (Appendix S1: Table S2): one observational, one aggregated, one literature, and three collections (Appendix S1: Table S2). This reduced the dataset from 10,836 entries to 1315 unique bee–plant‐source observations with 1066 unique bee–plant interactions across all sources in the normalized GloBI dataset (after all the filtering steps).
  4.4. *Formatting data for analysis*: To format the GloBI data for the analysis, we created a 3‐dimensional (3D) array with bee species as the first dimension (133 bee species), plant species as the second dimension (302 plant species), and source citation as the third dimension (six citations). This created an array with 240,996 possible bee–plant‐source combinations.
    We populated the 3D array with the observed bee–plant interactions for each source citation using the GloBI observations, where a value of one was used for each bee–plant interaction detected by each source citation and a value of zero for all non‐detections (i.e., possible bee–plant interactions based on the checklist but not documented).

### Model assumptions

Given that our use of GloBI data and application of an occupancy model were nontraditional, we explicitly outline several assumptions of the data and model:

1. We assumed that all bee and plant species occur across our entire geographic window. Thus, we assumed that bee–plant interactions were static and did not vary across space or time (i.e., no extinction or colonization events—a bee species always interacted with a plant species regardless of location and time).
2. We assumed that any bee species could potentially interact with any plant species, meaning we did not limit our analysis based on phenological or spatial overlap. Specifically, our formulation of the model is temporally aggregated, where Ѱ_*i*,*j*_ represents the probability that bee *i* and plant *j* have interacted at any point in time. We did not model intra‐annual phenology or interannual shifts explicitly. In earlier versions of our modeling, we attempted to estimate phenological overlap using bee flight periods from GBIF and flowering times from CalFlora, focusing on Santa Cruz Island and applying a geographic cutoff to account for spatial variation. However, this approach substantially reduced the size of our dataset. Many interactions observed on Santa Cruz Island were excluded when phenological filters were applied because the interactions fell outside of the bee flight periods from GBIF and/or flowering times from CalFlora, suggesting that the phenology estimates from these databases may not accurately capture local conditions. We also explored two additional strategies to estimate bee flight periods and plant flowering times: one using only local occurrence data and another approach integrating broader occurrence records with Santa Cruz Island weather data. The first approach lacked sufficient data coverage, with only 34% of bee species and 48% of plant species having more than three local records. The second approach, while promising in the plant phenology literature, requires dense occurrence data and often relies on average weather conditions, which may not reflect year‐to‐year variability, particularly in drought‐prone regions such as southern California (Mazer et al., 2015; Slade et al., 1975; Wang et al., 2022). Given these challenges, we determined that including phenology based on limited or potentially inaccurate data would introduce more bias than insight, and thus, we excluded phenological constraints from our final model to focus on the most reliable aspects of the dataset.
3. We assumed that each source citation had the opportunity to document all bee–plant interactions in our checklist. We inferred non‐detections for bee–plant interactions even when there was no evidence that each plant species was observed by a given source citation. In an attempt to verify that each source documented all bee–plant interactions, we searched the methods sections of primary literature and ensured that studies were bee community or interaction surveys, surveys documented all observed interactions, and that studies were conducted across a long period of flowering time. Our final six sources did in fact aim to record all bee–plant interactions. This allowed us to use the different source citations as “repeated surveys” for each bee–plant interaction (e.g., each source citation provides a detection/non‐detection replicate for that bee–plant interaction), like the approach used for double observer surveys. We assumed that collectors and observers would opportunistically collect or photograph all bee–plant interactions.
4. We assumed that all bee–plant interactions remained constant across observations (i.e., a bee with a high number of plant interactions would have a high number of interactions across all source types). Bees did not start interacting with new/different plant species, and since all bee–plant interactions occur across time, interactions account for the full suite of interactions or interaction changes from year to year.

The implications of these assumptions are discussed in *Discussion*: *Limitations*.

### Occupancy model formulation

We used an occupancy model to estimate the probability of bee–plant interactions and the total number of plant species that each bee species interacts with (Dorazio et al., 2006). We first defined bee species *i* interaction with plant species *j* (*z*_*i*,*j*_) as a binary variable, where *z*_*i*,*j*_ = 1 if bee species *i* interacts with plant species *j*, and 0 otherwise. The interaction state is assumed to be the outcome of a Bernoulli random process, where

(1) $$z_{i,j}\sim \text{Bernoulli}\left({Ѱ}_{i,j}\right).$$

Here, Ѱ_*i*,*j*_ is the probability that bee species *i* interacts with plant species *j* at any point in time. We did not model date‐specific interactions; thus, Ѱ_*i*,*j*_ is temporally aggregated. We included bee size, bee sociality, flower color, and flower shape as covariates in the bee–plant interaction model using a logit‐link function, where

(2) $$\text{logit}\left({Ѱ}_{i,j}\right)=\text{β1}+\text{β2}\times \mathrm{Bee}.{\text{size}}_{i}+\text{β3}\times \mathrm{Bee}.{\text{solitary}}_{i}+\text{β4}\times \text{Other}.\text{flower}.{\text{color}}_{j}+\text{β5}\times \text{Blue}.\text{flower}.{\text{color}}_{j}+\text{β6}\times \text{White}.\text{flower}.{\text{color}}_{j}+\text{β7}\times \text{Flower}.{\text{shape}}_{j}+u_{i}$$

β1 is the intercept term and represents the bee–plant interaction probability on the logit scale for an average size bee, social bee, yellow flower color, and not‐bowl flower shape. β2 is the slope coefficient between bee–plant interaction probability and bee size. We standardized bee size (covariate = Bee.size) by subtracting the mean and dividing by the SD. β3 is the added effect of nonsocial (i.e., solitary) bees. β4, β5, and β6 represent the added effect of other, blue, and white flowers, respectively, to the bee–plant interaction probability on the logit scale, where the variables Other.flower.color_*j*_, Blue.flower.color_*j*_, and White.flower.color_*j*_ are binary (0 or 1), with 1 representing the presence of the attribute for flower *j* and 0 otherwise. Similarly, β7 is the added effect of bowl‐shaped flowers to the bee–plant interaction probability on the logit scale, where the variable Flower.shape_*j*_ is binary (0 or 1), with 1 representing the presence of bowl shape for flower *j* and 0 otherwise. Lastly, we included a bee‐species random effect, *u*_*i*_, which was drawn from a normal distribution with mean, 0, and variance, σ_Ѱ_^2^, such that

(3) $$u_{i}\sim \text{Normal}\left(0,{\sigma_{Ѱ}}^{2}\right).$$

Next, we considered the detection process. The data, *y*_*i*,*j*,*k*_, consist of detection/non‐detection observations of bee species *i* on plant species *j* by source citation *k*. If bee species *i* is observed interacting with plant species *j* by source citation *k*, then *y*_*i*,*j*,*k*_ = 1 and 0 otherwise. We define the detection model as

(4) $$y_{i,j,k}\sim \text{Bernoulli}\left(p_{i,j,k}\times z_{i,j}\right)$$

where *p*_*i*,*j*,*k*_ is the probability of detecting the interaction of bee species *i* with plant species *j* by source citation *k* given that the bee–plant interaction does occur (*z*_*i*,*j*_ = 1). We model detection probabilities using a logit‐link function where

(5) $$\text{logit}\left(p_{i,j,k}\right)=\text{δ1}+\text{δ2}\times \mathrm{Bee}.{\text{strip}}_{i}+\text{δ3}\times \mathrm{Bee}.{\text{size}}_{i}+\text{δ4}\times \mathrm{Lit}.\text{citation}.{\text{type}}_{k}+\text{δ5}\times \mathrm{Col}.\text{citation}.{\text{type}}_{k}+\text{δ6}\times \mathrm{Agg}.\text{citation}.{\text{type}}_{k}+\text{δ7}\times \text{Other}.\text{flower}.{\text{color}}_{j}+\text{δ8}\times \text{Blue}.\text{flower}.{\text{color}}_{j}+\text{δ9}\times \text{White}.\text{flower}.{\text{color}}_{j}+\delta_{10}\times \text{Flower}.{\text{shape}}_{j}+v_{i}$$

δ1 is the intercept term and represents the bee–plant detection probability on the logit scale for an average size bee, not striped bee, observation data source type, yellow flower color, and not‐bowl flower. δ2 is the added effect of striped bees to the bee–plant detection probability on the logit scale, where Bee.strip_*i*_ is 1 when bee species *i* is striped and 0 otherwise. δ3 is the slope coefficient between bee–plant detection probability and bee size. Again, we used standardized bee size (Bee.size) by subtracting the mean and dividing by the SD. δ4, δ5, and δ6 represent the added effect of literature, collections, and aggregated sources, respectively, to the bee–plant detection probability on the logit scale, where the variables Lit.citation.type_*k*_, Col.citation.type_*k*_, and Agg.citation.type_*k*_ are 1 when source type *k* is literature, collections, and aggregated sources, respectively, and 0 otherwise. As before, δ7, δ8, and δ9 represent the added effect of other, blue, and white flowers, respectively, to the bee–plant detection probability on the logit scale, and δ10 is the added effect of bowl‐shaped flowers to the bee–plant detection probability on the logit scale. Lastly, we included a bee‐species random effect, *v*_*i*_, which was drawn from a normal distribution with mean, 0, and variance, σ_*p*_^2^, such that

(6) $$v_{i}\sim \text{Normal}\left(0,{\sigma_{p}}^{2}\right).$$

To estimate the total number of unique bee–plant interactions (*N*_*i*_), including the number of species interactions not observed (but present) during sampling, we summed *z*_*i*,*j*_ across plant species 1:J to calculate the total number of plant species that each bee species interacts with.

In addition to the bee‐species random effect occupancy model presented here in the main text, we ran several other models to explore broader phylogenetic constraints on interactions (Appendix S2). Specifically, in Appendix S2, we present the following additional occupancy models and results with random effects at the bee family, plant family, bee and plant family, plant species, and no random effect.

### Parameter comparisons

For categorical variables, to quantify differences among pairs of parameter estimates, following Ruiz‐Gutiérrez et al. (2010), we computed the proportion of iterations where the reference‐level parameter (e.g., *a*) was greater than the sum of the reference‐level and categorical effect parameters (e.g., *a + b*), written throughout as Pr(*a* > *b*) for brevity. This can be directly interpreted as the probability that the first parameter is greater than the second. Values close to zero suggest that the first parameter is lower than the second parameter, whereas values close to one suggest that the first parameter is higher than the second parameter. Values near 0.50 suggest that both parameter estimates are similar (i.e., no difference).

For continuous variables, we calculated the proportion of the posterior distribution that was above or below zero. This value can be interpreted as the probability that the parameter is greater or less than zero. Values close to either zero or one suggest a difference, whereas values near 0.50 suggest that the parameter estimate is similar to zero (i.e., no difference).

### Effect sizes

To quantify the magnitude of difference between model parameters, we calculated predicted probabilities at ecologically meaningful values. For continuous variables, we computed interaction or detection probabilities at the lowest and highest observed values of the data while holding other variables at their reference levels (i.e., social bees, yellow flowers, not‐bowl‐shaped). For categorical variables, we calculated predicted probabilities for each category with other variables held at their means (for continuous variables) or reference levels (for categorical variables). Effect sizes were expressed as the absolute difference in predicted probabilities between comparison groups. We report these differences along with their 95% credible intervals derived from the posterior distribution.

### Model fitting

We analyzed the dataset using a Bayesian approach in the nimble package in R (de Valpine et al., 2017, 2022). The model was run on the U.S. Geological Survey (USGS) Hovenweep supercomputer, accessed through the USGS Advanced Research Computing facility (Falgout et al., 2025). We ran the model for 250,000 iterations with a burn‐in of 50,000 iterations and thinning by 10. We ran the model with a total of three chains. For the covariate parameters, we used priors using a mean of zero and SD of one. For the variance parameters, we used a truncated normal distribution with mean zero and SD of two, truncated at zero with no upper limit. We assessed convergence using the Ȓ statistic (Brooks & Gelman, 1998) and visually inspected traceplots. We did not conduct a goodness of fit test because goodness‐of‐fit tests may indicate poor fit in cases with small sample size (DiRenzo et al., 2023).

### Interaction networks

To understand the difference between the bee–plant interactions predicted from the occupancy model and the normalized GloBI dataset, we calculated network metrics, such as structure and specialization, from each bipartite network. These networks are based on visitation data and do not imply that the visited plant necessarily serves as the pollen host for the bee species, or that the plant is pollinated by the bee visitor. For the normalized GloBI dataset, we incorporated all bee–plant interactions to build a visitation network.

#### Occupancy model network

From the occupancy model output, we used the variable *z*_*i*,*j*_ (i.e., a binary variable that indicates if bee species *i* interacts with plant species *j*) and calculated the average across Markov chain Monte Carlo (MCMC) iterations. This resulted in a *z*_*i*,*j*_ for each bee and plant species combination between the value of 0 and 1. As all interactions were possible in the model output, some values such as nestedness were no longer calculable. Thus, we limited the data for the network calculation and visualization and explored a range of cutoff values for *z*_*i*,*j*_ between 0 and 1 (i.e., every 0.10) to determine how the cutoff value affected the resulting bipartite network. For the bipartite visualization and calculated network metrics of the occupancy model output presented in the text, we limited our bee–plant networks to *z*_*i*,*j*_ values of 0.50 or greater. The value for *z*_*i*,*j*_ was then multiplied by 10 to simulate a number of interactions between a given bee species and plant species within the network matrix.

#### Network metrics

For the bipartite network generated by the normalized GloBI dataset, we calculated metrics of structure (i.e., connectance and nestedness based on overlap and decreasing fill [NODF]) and specialization (i.e., H_2_′ and interaction evenness) using the package *bipartite* and function *networklevel* (Dormann et al., 2008, 2009). Connectance is the proportion of possible links observed in a network ranging from 0 (low connectance) to 1 (high connectance). Nested structure or asymmetry of interactions was measured using the NODF (Almeida‐Neto et al., 2008) which ranges from 0 (low nestedness) to 100 (high nestedness). H_2_′ measures reciprocal specialization between interaction partners and ranges from 0 (low specialization) to 1 (high specialization) and interaction evenness is measured with Shannon's evenness of interactions ranging from 0 (low evenness) to 1 (high evenness). To determine if each metric calculated for the GloBI network was significantly different from that of a random network structure, we compared the normalized GloBI network to null networks (*n* = 500). Null networks were randomly generated with the same connectance as the input network using the function, *nullmodel* (Vázquez et al., 2007). We then calculated the z‐score of each network metric to determine if the structure and specialization of the GloBI network was significantly different from the randomized network structure (*p* < 0.05). We then repeated the same steps for calculating network metrics for the model output network.

All data and code to reproduce the analyses for this manuscript are available on two Zenodo repositories (Lee et al., 2025; Seltmann et al., 2025) and the GitLab repository (Lee et al., 2026), respectively.

## RESULTS

### Checklist composition and sample sizes

Our final bee species checklist totaled 133 species, which included 121 solitary bee species and 12 social bees. Our bee species checklist also included 66 striped species and 67 nonstriped species. The median bee size was 8.44 mm (min = 2.54 mm [*Lasioglossum hammondi*]; max = 16.5 mm [*Bombus crotchii*]).

Our final plant species checklist totaled 302 species, which included 87 species with yellow flowers, 58 blue/purple flowers, 94 white flowers, and 54 other colored flowers, which included pink, green, orange, and red. Bowl‐shaped flowers made up 197 species in our checklist and not‐bowl‐shaped flowers made up 105 species.

### Total number of observed and estimated interactions

From the raw bee–plant data (i.e., observed GloBI database), there were 1066 unique bee–plant interactions documented across all sources. Of those, the maximum number of observed plant interactions per bee species was 124 by *Bombus vosnesenskii*, and the average number of plant species that each bee species interacts with was 8.01 (SE = 1.57, range = 0–124; Appendix S1: Figure S1). Fifty‐two of the 133 checklist bee species had 0 plant species interactions documented in the GloBI database, 12 bee species had only one plant species interaction, and 10 bee species had only two plant species interactions documented (Appendix S1: Figure S1).

In contrast, we found that the occupancy model predicted that the number of plant species that each bee species interacted with ranged from 11 to 262 (mean = 103.67, SE = 5.81; Appendix S1: Figure S1). We report all parameter estimates from the bee‐species random effect occupancy model in Table 1.

**TABLE 1:** Summary of parameter estimates from the bee‐species random effect occupancy model.

Parameter definition	Symbol	Mean	SD	95% credible intervals	
Intercept of bee–plant interaction probability on logit scale which refers to an average size bee, social bee, yellow flower color, and not‐bowl‐shaped flower	β1	−0.76	0.83	−2.48	0.76
Bee size	β2	−0.40	0.40	−1.13	0.49
Bee solitary	β3	0.54	0.74	−0.94	1.97
Other flower color	β4	0.36	0.29	−0.21	0.94
Blue flower color	β5	0.86	0.24	0.39	1.34
White flower color	β6	−0.26	0.22	−0.69	0.17
Bowl flower shape	β7	−1.83	0.35	−2.54	−1.17
Intercept of bee–plant detection probability on logit scale: an average size bee, not striped bee, observation source type, yellow flower color, and not‐bowl‐shaped flower	δ1	−4.92	0.41	−5.80	−4.16
Bee stripes	δ2	0.06	0.38	−0.69	0.77
Bee size	δ3	0.57	0.25	0.07	1.07
Literature source type	δ4	−1.40	0.10	−1.59	−1.20
Collection source type	δ5	−1.13	0.07	−1.27	−1.00
Aggregated source type	δ6	−1.45	0.10	−1.65	−1.25
Other flower color	δ7	−0.48	0.16	−0.79	−0.16
Blue flower color	δ8	0.11	0.12	−0.14	0.34
White flower color	δ9	0.17	0.13	−0.08	0.42
Bowl flower shape	δ10	1.15	0.11	0.92	1.36
SD of bee species detection probability	σ_*p*_^2^	1.70	0.25	1.25	2.24
SD of bee species interaction probability	σ_Ѱ_^2^	2.61	0.61	1.53	3.88

*Note*: We include parameter definitions, symbols, mean and SD of the posterior distributions, and the 95% credible intervals. All β and δ values are on the logit scale.

### Ecology of bee–plant interactions

**FIGURE 1:**
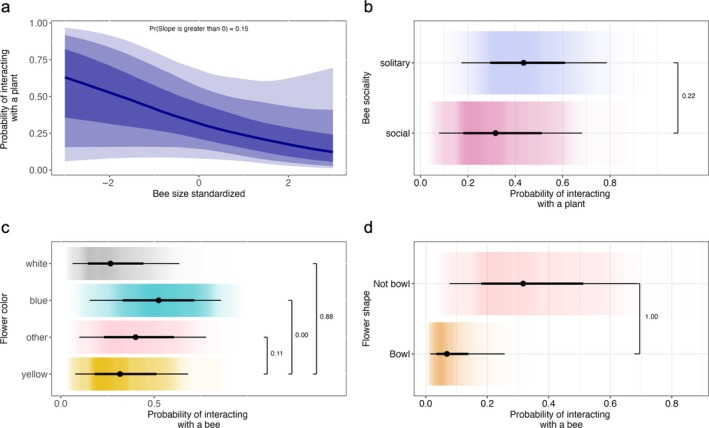
Bee–plant interaction probabilities as a function of the following covariates (a) bee size, (b) bee sociality, (c) flower color, and (d) flower shape. The shaded regions in panel (a) display the 50%, 80%, and 95% credible intervals (from darkest to lightest shading), derived from the posterior distribution of the Markov chain Monte Carlo samples, with the solid line indicating the posterior mean. In panels (b) to (d), the black dot is the posterior mean and the thick and thin horizontal lines are the 66% and 95% credible intervals, respectively. The color gradient backgrounds are kernel‐smoothed densities of the posterior samples, with darker color indicating where the distribution is most concentrated. Horizontal brackets link the two categories being contrasted; the number printed at the bracket's right‐hand side is the posterior probability that the reference category has a higher interaction probability than the category to which it is connected.

We found a 0.85 probability that interacting with a plant was negatively related to bee size (i.e., 85% of the posterior distribution for β2 was negative; Figure 1a), where larger bees had lower probabilities of interacting with a plant than smaller bees. Specifically, small bees (2.54 mm) had a 0.53 (95% CI = 0.07–0.94) probability of interacting with plants, while large bees (16.5 mm) had a 0.17 (95% CI = 0.009–0.71) probability, a difference of 0.36 (95% CI = −0.50 to 0.87).

We did not find evidence that social bees had a higher probability of interacting with plants than solitary bees (Pr(β1 > β3) = 0.22; Figure 1b). Solitary bees had a 0.44 probability of interacting with plants (95% CI = 0.17–0.78), while social bees had a 0.33 probability (95% CI = 0.07–0.68), an average difference of 0.10 (95% CI = −0.21 to 0.41).

We found that flower color influenced the probability of bee–plant interactions. Specifically, yellow, white, blue/purple, and other colored flowers had a 0.33 (95% CI = 0.07–0.68), 0.29 (95% CI = 0.06–0.63), 0.52 (95% CI = 0.15–0.85), and 0.41 (95% CI = 0.09–0.77) probability of interacting with a bee species, respectively. When comparing these values pairwise, yellow flowers had a 0.04 (95% CI = 0.00–0.15) higher bee interaction probability compared to white flowers (Pr(β1 > β6) = 0.88), and yellow flowers had a 0.18 (95% CI = 0.06–0.30) lower bee interaction probability than blue/purple flowers (Pr(β1 > β5) = 0.001). However, yellow flowers had a similar bee interaction probability as other colored flowers (Pr(β1 > β4) = 0.11; Figure 1c).

Lastly, we found that not‐bowl‐shaped flowers had a higher bee interaction probability than bowl‐shaped flowers (Pr(β1 > β7) = 1.00; Figure 1d). Not‐bowl‐shaped flowers had a 0.33 (95% CI = 0.07–0.68) probability of interacting with bees, while bowl‐shaped flowers had a 0.08 (95% CI = 0.01–0.25) probability, a difference of 0.25 (95% CI = 0.06–0.46).

### Detection of bee–plant interactions

**FIGURE 2:**
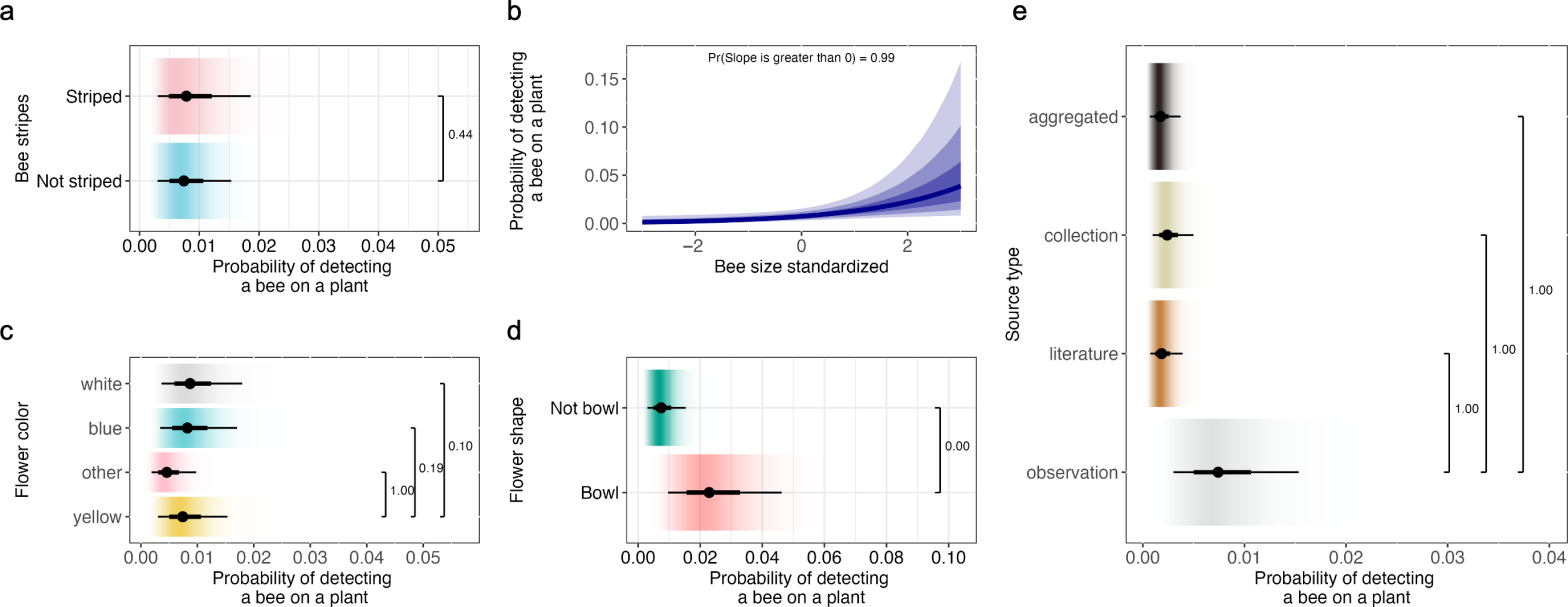
Bee–plant detection probabilities as a function of the following covariates (a) bee stripes, (b) bee size, (c) flower color, (d) flower shape, and (e) source type. The shaded regions in panel (a) display the 50%, 80%, and 95% credible intervals (from darkest to lightest shading), derived from the posterior distribution of the Markov chain Monte Carlo samples, with the solid line indicating the posterior mean. In panels (b) to (e), the black dot is the posterior mean and the thick and thin horizontal lines are the 66% and 95% credible intervals, respectively. The color gradient backgrounds are kernel‐smoothed densities of the posterior samples, with darker color indicating where the distribution is most concentrated. Horizontal brackets link the two categories being contrasted; the number printed at the bracket's right‐hand side is the posterior probability that the reference category has a higher interaction probability than the category to which it is connected.

Striped bees had a 0.008 (95% CI = 0.003–0.01) probability of being detected on a plant, and not striped bees had a 0.007 (95% CI = 0.003–0.01) probability of being detected on a plant. We did not find that striped bees had a higher detection probability than not striped bees (Pr(δ1 > δ2) = 0.43; Figure 2a). However, we found that bee detection probability was related to bee size (Pr(δ3 > 0) = 0.98; Figure 2b), where larger bees had a 0.05 (95% CI = 0.008–0.20) detection probability and smaller bees had a 0.002 (95% CI = 4e−04–0.008) probability of being detected.

We found that flower color had a large impact on bee–plant detection probability, where yellow, white, blue/purple, and other colored flowers had 0.007 (95% CI = 0.003–0.015), 0.009 (95% CI = 0.003–0.017), 0.008 (95% CI = 0.003–0.017), and 0.004 (95% CI = 0.001–0.009) probabilities of having a bee detected on them, respectively. Bees were 0.002 (95% CI = 0.0007–0.006) more likely to be detected on yellow flowers than other colored flowers (Pr(δ1 > δ7) = 0.99; Figure 2c), but bees were 0.001 (95% CI = −7.10e−04 to 0.004) more likely to be detected on white flowers than yellow flowers (Pr(δ1 > δ9) = 0.09; Figure 2c) and bees were 0.0008 (95% CI = −0.001 to 0.003) more likely to be detected on blue/purple flowers than yellow flowers (Pr(δ1 > δ8) = 0.19; Figure 2c).

We also found that bees on bowl‐shaped flowers had a higher detection probability than bees on not‐bowl‐shaped flowers (Pr(δ1 > δ10) = 0.00; Figure 2d). Bees on bowl‐shaped flowers had a 0.02 (95% CI = 0.009–0.04) probability of being detected, while bees on not‐bowl‐shaped flowers had a 0.007 (95% CI = 0.003–0.015) probability of being detected, a difference of 0.01 (95% CI = 0.006–0.031).

Lastly, literature, collections, aggregated, and observation sources had 0.001 (95% CI = 0.0007–0.003), 0.002 (95% CI = 0.0009–0.004), 0.001 (95% CI = 0.0007–0.003), and 0.007 (95% CI = 0.003–0.015) probabilities of detecting a bee–plant interaction, respectively. We found that observation source types had a higher probability of detecting bee–plant interactions than any other source type (e.g., aggregate, collection, literature; Pr(δ1 > δ4) = 0.00; Pr(δ1 > δ5) = 0.00; Pr(δ1 > δ6) = 0.00; Figure 2e), where observations had a 0.006 (95% CI = 0.002–0.011) higher probability of detecting a bee–plant interaction than aggregated sources, a 0.005 (95% CI = 0.002–0.010) higher probability of detecting a bee–plant interaction than collections, and a 0.005 (95% CI = 0.002–0.011) higher probability of detecting a bee–plant interaction than literature.

### Interaction networks

**FIGURE 3:**
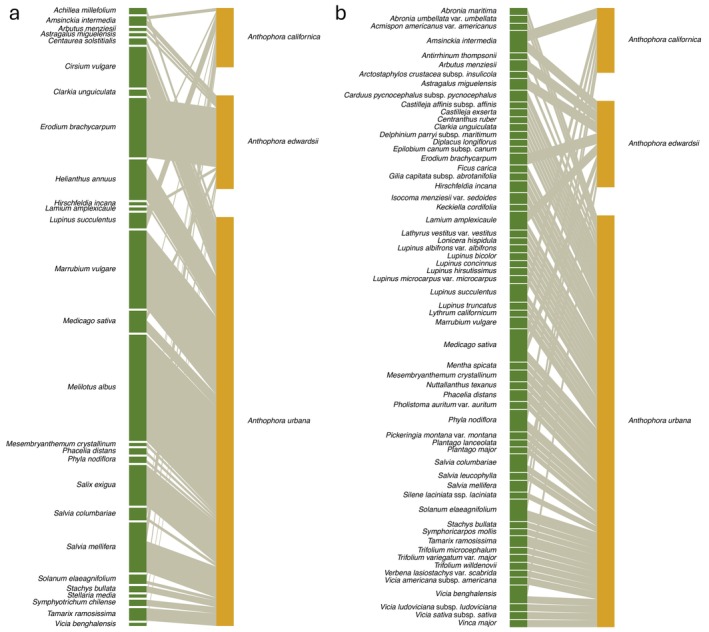
Bee–plant interaction bipartite networks for the bee genus *Anthophora* (Apidae) based on (a) the Global Biotic Interactions (GloBI) and (b) model point estimate network output. Larger bees, like *Anthophora* and *Bombus*, had a lower probability of plant interactions compared to smaller bees. The networks visualized here are limited for the sake of readability. Network visualizations have plants on the left‐hand side and bee species on the right‐hand side. The modeled network visualized here accounts for all interactions with a *z*_*i*,*j*_ value of 0.50 or higher. The plants included in the occupancy model bipartite network match those within the GloBI dataset. Lines between bees and plants indicate interactions and the thickness of those lines represents the frequency or *z*_*i*,*j*_ value of those interactions. Network visualizations for the bee family Apidae are presented in Appendix S3.

The GloBI network and the occupancy model output network differed across all network measures of interest when compared visually (Appendix S1: Table S3). Across values of modeled interaction (*z*_*i*,*j*_) cutoffs, connectance, interaction evenness, and NODF values of the model output network were higher than that of the GloBI network and reciprocal specialization (H_2_′) was lower (Appendix S1: Figure S2). After limiting the modeled network to interactions with a cutoff value of 0.50 or higher (Figure 3), we found that almost all network metrics generated from both the occupancy model output and the normalized GloBI interactions (i.e., interaction evenness, nestedness, and specialization) were significantly different compared to null networks, indicating that these network patterns are nonrandom (Appendix S1: Table S3). Only reciprocal specialization (H_2_′) of the occupancy model output network was not significantly different compared to null networks. When comparing the occupancy model network with the GloBI network, we found that interaction evenness (occupancy model output network: 0.912 vs. GloBI network: 0.543) and connectance (0.417 vs. 0.066) of the occupancy model output network were higher than those of the GloBI network. The modeled network had a higher measure of nestedness relative to the GloBI network using NODF (92.704 vs. 30.954).

## DISCUSSION

In this study, we explored several hypotheses related to bee and plant characteristics that may drive ecological and detection processes within species interaction networks. We found that bee–plant interactions in our ecological model were best predicted by bee size, flower color, and flower shape, and that bee–plant detections were best predicted by source type, bee size, and flower color and shape. We found that the bipartite network generated by the occupancy model output was more even, nested, and specialized relative to the network generated by GloBI data alone. These results reflect some of our broader understandings of bee–plant interactions and highlight detection biases that may influence our ability to understand species interactions.

### Ecological effects

We did not find that social bees had a higher probability of interacting with plants than solitary bees, despite some evidence that social bees that forage collectively may be able to visit more floral partners than solitary bees (Araújo et al., 2021; Bogusch et al., 2006; Kaluza et al., 2017, 2018). This finding related to bee sociality may reflect the Santa Cruz Island checklist that largely consists of solitary bee species (91%). We found that larger bees (greater than 8.5 mm, including *Bombus* and *Anthophora*) had a lower probability of plant interactions compared to smaller bees (less than 8.5 mm, including *Lasioglossum* and *Hylaeus*). While this result contrasts with studies that have shown that larger bees have a proportionally larger foraging range and associated diet breadth (Cullen et al., 2021; Greenleaf et al., 2007), research on smaller bees is limited and more recent studies indicate that their foraging ranges may be larger than expected based on size alone (Dorey et al., 2024; Kline et al., 2022; Nelson et al., 2023). Furthermore, generalist species such as *Bombus* may exhibit more specialized foraging preferences than their size would suggest, as their realized foraging habits are influenced by inherent preference (Wood et al., 2021). Finally, this result may reflect the tendency for island bees to be more generalized in their diet or visitation (Traveset et al., 2016). The absence of a geographic cutoff in our data might underscore the dietary flexibility of common small bees that are able to adapt to many geographic regions by exploiting different floral resources. It is also important to note that our dataset consisted of bee–plant interactions and visitation records that were not solely restricted to pollination events; therefore, our results may reflect bee behaviors or plant visitation beyond pollen collection or plant reproduction.

Flower color and shape were both important covariates for predicting bee–plant interactions. Flower traits in other systems have been identified as important covariates to consider in plant–pollinator interactions (Bartomeus, 2013; Olesen et al., 2007; Ornai & Keasar, 2020; Rowe et al., 2020). Blue/purple flowers were most likely to have a bee interaction relative to yellow and white flowers, which aligns with other bee studies that show bees have a strong preference for blue/purple (Leong & Thorp, 1999; Lunau & Maier, 1995; Saunders & Luck, 2013), despite yellow and white flowers also being considered bee attracting colors and generally pollinator‐attracting colors. We recognize that our classification of flower color is based on human vision, and bee vision also includes the ultraviolet range that we could not include here (Gumbert et al., 1999). Similarly, we chose to include what could have been classified as purple flowers into our blue flower grouping as these colors were not easily distinguished during our classification. However, we recognize that these colors vary in their spectral reflectance and think the classification of coloration as it is seen by bees could be improved in future analyses.

Lastly, we found that not‐bowl‐shaped flowers had a higher probability of a bee interaction than bowl‐shaped flowers, despite bowl‐shaped flowers making up 65% of our plant checklist. Olesen et al. (2007) found no association between flower openness and visitor generalization. Based on field studies, a preference for not‐bowl‐shaped flowers may be a common trend among solitary bees, which make up the vast majority of the bee species in our checklist (Sponsler et al., 2022; Wang et al., 2024). Though these flowers may have greater physical barriers to floral rewards, these rewards may offer higher quality resources such as nectar or pollen (Olesen et al., 2007).

### Detection

Our detection model results reflected observer behavior (both collections and community scientists), where community science observers (i.e., observers using iNaturalist) were more likely to observe an interaction than a specimen collector, reflecting the more opportunistic behavior of community scientists, who may record and upload any encountered interactions. Conversely, more discerning, or goal‐oriented collectors record and collect unique interactions that address specific scientific or collection‐based questions (Meineke & Daru, 2021). This may also be the result of our inclusion of only citations with over 99 records, leaving the bulk of records from community scientist data and removing many scientific or collection‐based records. In future analyses it may be important to experiment with citation groupings to try to retain these records. Broadly, the variability in detection probability, influenced by the data source, highlights how combining these sources can enrich our understanding of interaction networks, providing insights that would be missed if relying on a single type of data.

As community scientist observations made up the bulk of our data, this likely impacted detection covariates, such as the influence of flower coloration and bee size. Larger bees are much easier to observe, and this can bias our understanding of bee–plant interactions. The same may be true for flower color and shape, as bowl‐shaped flowers are easier to observe a bee–plant interaction than a not‐bowl‐shaped flower.

We did not find that yellow flowers were more likely to be observed relative to blue/purple flowers; in fact, bees on blue/purple and white flowers were more likely to be observed than bees on yellow flowers. Further, we did not find that striped bees were more likely to be detected than nonstriped bees. These findings do not align with our hypotheses, which predicted that traits making plants and bees more conspicuous would enhance their detectability. Interestingly, since the majority of citations in the data came from iNaturalist observers, it is possible that these observers had a greater understanding related to the nature of bee–plant interactions more broadly.

These findings related to the effect of floral covariates on detection probability may be due to the coarseness of the scale of our analysis. If we had a larger sample size, we would have parsed out flower color to all the different colors (i.e., pink, red, orange, green), and we would have included a larger variety of flower shapes. However, given the sample size of our dataset, adding more covariates to the model was not possible.

Lastly, we want to highlight the low bee–plant detection probabilities reported in this study. Even with an occupancy framework, fragmented spatiotemporal coverage and heterogeneity in sampling among sources translate incomplete sampling of true bee–plant interactions, particularly for small‐bodied or narrowly distributed taxa. Accordingly, we emphasize the direction and relative magnitude of effects (e.g., traits and source types that systematically elevate detection and interaction odds) over absolute probabilities or total number of species interactions, which had high levels of uncertainty in some cases (Appendix S1: Figure S1). The patterns of effects from species traits and sources are robust to underdetection, whereas absolute values are expected to be biased downward.

### Network structure

With additional predicted interactions, the interaction network generated from the occupancy model output was more even, nested, generalized than we would predict with observed interactions alone. Additional trait data revealed possible interactions that were unobserved by collectors due to interaction rarity, species rarity, or observer biases, which in turn increased network connectance and evenness. Thus, it is possible that our modeled network could improve our understanding of interactions for bee and plant species that are not often observed (i.e., rare species).

Contrary to our expectations, we found that the occupancy model network was more nested than the GloBI network and was significantly nested relative to null or random network structure. As greater nestedness, generalization, and evenness reflect greater network stability in the context of environmental perturbations and species loss (Duan et al., 2023; Waser & Ollerton, 2006), supplemented interactions from modeled networks may reflect a more robust structure of species interactions than the observed network. However, as we assumed that all interactions were possible, it is likely that we overestimated the number of possible bee–plant interactions as we did not take “forbidden links” into account that could have been due to trait or phenological mismatches (Olesen et al., 2010). We expect that the true interaction network structure of Santa Cruz Island bee and plant species lies somewhere in between the observed (i.e., GloBI) and modeled (i.e., occupancy output) networks presented here.

### Limitations

Adding covariates to our ecological and detection models improved our understanding of both ecological and data collection (detection) processes. However, we were limited by the number of covariates we could include and estimate for two reasons. First, the number of observations in the final dataset (*n* = 1066 unique bee–plant observations) was low given the number of possible bee–plant interactions we were trying to estimate (*n* = 40,166 possible bee–plant interactions). While observations of interactions are generally increasing with data aggregation and digitization efforts, the lack of ecological data for the majority of the community contributed to the low detection probabilities and high levels of uncertainty in estimating the bee–plant interactions (Appendix S1: Figure S1). Second, hierarchical Bayesian models require a lot of computational power and can take long periods of time to run (Yackulic et al., 2020). Different iterations of our model took between two and five days to run on a supercomputer. Future studies could work toward integrating more data and adding more covariates, potentially leveraging valuable data regarding bee lecty or diet specialization to add specific probability scores to each species interaction and to remove forbidden links and move these interaction data more in the direction of pollination. However, as these data currently stand, less than 50% of North American bees have recorded diet data (Smith et al., 2024). Future consideration of trait covariates and interaction mismatches will be a fruitful avenue for exploration. Including phenology estimates based on yearly weather and/or flight timing, increasing data (number published interaction observations) and abundance data could improve the predictive ability, including network predictions based on yearly variation or future climate change.

We also acknowledge that some of the assumptions we declared of the data and occupancy model above impact our inference. Specifically, we were unable to include either species phenology or range size in a meaningful way in the analysis due to limited data availability (explained above). Because species phenology and range size are unmodeled in our analysis, we anticipate that species with longer activity windows (flowering/flight periods) and/or larger spatial ranges will have higher interaction probabilities (ψ; i.e., interact with more species) and/or higher detection probabilities (*p*; i.e., easier to detect), while species with narrower activity windows and/or ranges will appear to be ecologically rare or difficult to detect. Longer activity windows and larger spatial ranges primarily impact a species availability, not their interaction or detection probabilities. Therefore, as more data become available, future iterations of this modeling work may consider accounting for species phenology and/or range size using multi‐scale occupancy models (DiRenzo et al., 2022; Nichols et al., 2008).

## CONCLUSIONS

Throughout this work, we discovered multiple avenues in which the study of interactions using both occupancy modeling and aggregated datasets can be improved to better estimate ecological and detection probabilities. Data may be more reusable if published records of interactions include geolocality information and individual observations rather than summary tables of species‐level observations. Our work also highlights the value of species checklists that leverage both the expertise and time of professional collectors and taxonomists. Taxonomic identification and observation of rare species can be difficult (Meiners et al., 2019), and taxonomic names require consideration and an understanding of taxonomic nomenclature. Non‐peer‐reviewed species checklists cited as independent research are important sources of data, even though they do not carry the formal authority conferred by peer review. We encourage authors to publish them as journal articles.

Observations of species interactions dictate our ability to both predict and protect ecosystem structure and function. Indeed, bee–plant interactions, specifically pollination, provide great service to whole ecosystems through the ensured persistence of plant populations that create habitat and food resources for all trophic levels. These interactions are increasingly threatened, and researchers' ability to understand our own biases that shape our understanding of these interactions will prove vital in our pursuit of conservation action. Moreover, our understanding of many kinds of species interactions can be improved by utilizing a similar modeling approach. Our study is among the first of its kind to utilize occupancy modeling to directly model species' interactions, leveraging both aggregated, open‐source databases and expert checklists. Within the last 2 years, bee interaction data available on GloBI has increased by over half a million records (Seltmann et al., 2025), and iNaturalist.com just reached 200 million verifiable species observations as of July 2024 (Loarie, 2024). Our findings stress the importance of investigating the effect of detection and collection biases on our understanding of ecological processes. This work has the potential to further our understanding of biological interactions more broadly, leveraging newly populated and rapidly available datasets and methods.

## AUTHOR CONTRIBUTIONS

Katja C. Seltmann conceived of the project. All coauthors contributed to project and model development. Katja C. Seltmann contributed to GloBI database development. Michelle J. Lee, Chengyi Diao, and Katja C. Seltmann contributed to trait dataset development. Graziella V. DiRenzo and Michelle J. Lee formatted and normalized data. Graziella V. DiRenzo wrote the model. Graziella V. DiRenzo and Michelle J. Lee analyzed data. Michelle J. Lee, Graziella V. DiRenzo, and Katja C. Seltmann wrote the first draft of the manuscript. All coauthors edited the manuscript.

## CONFLICT OF INTEREST STATEMENT

The authors declare no conflicts of interest.

## Supporting information


Appendix S1.

Appendix S2.

Appendix S3.

## Data Availability

Data are available in the following Zenodo releases: Seltmann, Poelen, and Global Biotic Interaction Community (2025), https://doi.org/10.5281/zenodo.17957582; Lee et al. (2025), https://doi.org/10.5281/zenodo.13539345. Code (Lee et al., 2026) is available in GitLab at https://doi.org/10.5066/P9ZMJ9YD.
