# Supplementary material for: Leveraging local species data, a global database, and an occupancy model to explore bee–plant interactions
Source: Ecol Appl. 2026 Mar 24;36(2):e70221. doi: 10.1002/eap.70221 (PMC13012871; doi:10.1002/eap.70221)
Supplement: Supplementary file 1 — Appendix S1. [file EAP-36-e70221-s001.pdf]

Appendix S1

Leveraging local species data, a global database, and an occupancy model to explore bee–plant interactions

Michelle J. Lee, Graziella V. DiRenzo, Chengyi Diao, Katja C. Seltmann

Ecological Applications

Any use of trade, firm, or product names is for descriptive purposes only and does not imply endorsement by the U.S. Government.

Appendix S1: Methods

Compiling trait data

Bees- For all bee species on the checklist, bee size was obtained from the original descriptions as recorded on the Discover Life website (https://www.discoverlife.org/mp/20q?guide=Apoidea_species&flags=HAS) or from publications (listed in Lee et al. 2025). Bee size was recorded as a range for both males and females when both were available. We used minimum female size for the analyses because the body size sex differences were generally correlated and body size metrics for males were unknown for many species.

Bee sociality was documented using "The Bees In Your Backyard" (Wilson & Carril, 2015) including broad categories as defined in the book: eusocial, semisocial, communal, nest aggregation, solitary, and parasitic. Social bees include those bees that are eusocial, and those that are primitively or facultatively eusocial (Danforth et al., 2019; Ostwald et al., 2024). Social status was largely inferred at the genus level, except within the Halictidae where species-level was investigated due to the high amount of variation, especially within the *Lasioglossum* genus. In cases where the species of *Lasioglossum* sociality was not known, we assumed solitary except those in the subgenus *Hemihalictus* because the majority of the non-*Hemihalictus Lasioglossum* bees are considered solitary (Brady et al., 2006; Soucy, 2002).

To quantify bee coloration, we captured full-text descriptions from the literature for both male and females. We generalized from the text to describe several color patterns found on the three body segments (head, mesosoma, metasoma). The color patterns were coded as binary with 1 representing true or 0 representing false for stripes, dark coloration, metallic, or patterned. Patterned is defined as broad colored areas that do not create repeated, uniform stripe patterns. We also used images from Discover Life (Ascher & Pickering, 2020), Bee Library (Seltmann et al., 2021), BugGuide, (Global Biodiversity Information Facility) GBIF and iNaturalist to check these trait characterizations. Only striped abdomen, and patterned abdomen were combined in the model reflecting high contrasting color patterns.

Plants- For each plant species, we collected data related to floral color (i.e., yellow, blue/purple, white, etc.) and floral shape (i.e., bowl shaped corolla or not) using Calflora and Jepson eFlora (*Jepson Flora Project (Eds.)*, 2022). Floral shape refers to the accessibility of floral resources to pollinator species and follows the designation by Bosch et al., (1997), Bartomeus et al., (2013), and Olesen et al., (2007).

In order to test our hypothesis about the effect of floral shape on bee-plant interaction probabilities and detection probabilities, we classified flowers as bowl versus not-bowl shape. Bowl shaped flowers included dish and brush shapes whereas not-bowl shaped flowers included bells, tubes, and flower shapes with gullets or flags. We used groupings by Olesen et al. (2007), but we based categorization for this paper on a broad suite of organisms' ability to access the floral resources and an observer's ease of detecting an interaction. We decided to keep these descriptors (i.e., bowl versus not-bowl) within these broad categories as we did not want to use a term that might mischaracterize the flower's morphology.

Appendix S1: Table S1. Various flower shape terms, the corresponding category (i.e., not-bowl versus bowl shape) that was used in this study, and a brief description of the floral shape.

Flower shape term	Category	Brief description
Bell, campanulate	Not-bowl shape	Bell-like, downward-facing tubular structure
Tube/Tubulate	Not-bowl shape	Long, narrow tube or trumpet form
Dish	Bowl shape	Wide, open, concave petals forming a bowl-like structure
Brush	Bowl shape	Open blossoms with long and sometimes dense stamens and styles
Gullet	Not-bowl shape	Chamber shape that pollinator must enter
Flag	Not-bowl shape	Restricted access to stamen with wings, keel, and banner petals, papilionaceous
Cyanthiform	Bowl shape	Cup or bowl shaped; also cruciform, cateriform, stellate
Coronate	Not-bowl shape	Crown-like flower
Calceolate	Not-bowl shape	Shoe or slipper-like
Urceolate	Not-bowl shape	Urn shaped
Salverform	Not-bowl shape	Long tube with a flat, spreading limb
Saccate	Not-bowl shape	Tube with disc of petals at the bottom
Rotate	Bowl shape	Wheel like with unfused petals
Ligulate	Bowl shape	Strap-shaped petals arranged in an open plane
Labiate/Bilabiate	Not-bowl shape	Combines tubular and two-lipped features
Galeate	Not-bowl shape	Hooded or helmeted

All covariate data files and bee trait references can be found on Zenodo (Lee et al., 2025).

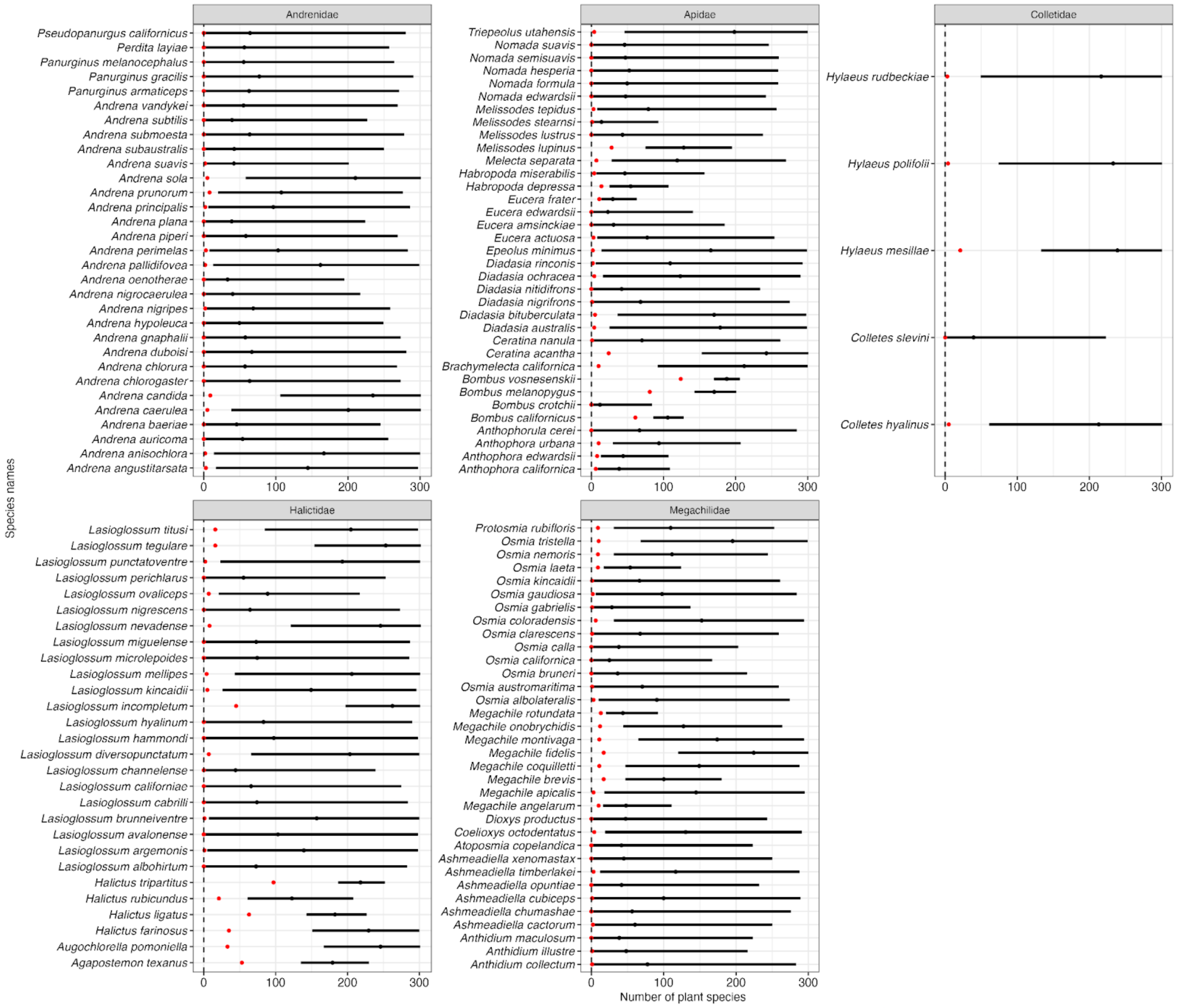

Appendix S1: Figure S1. Number of plant interactions per bee species from the observational data (red points), and the number of plant interactions per bee species estimated by the occupancy model (black points = mean; line range = 95% credible interval) faceted by bee family. Bee species are listed in alphabetical order starting at the bottom within family panel.

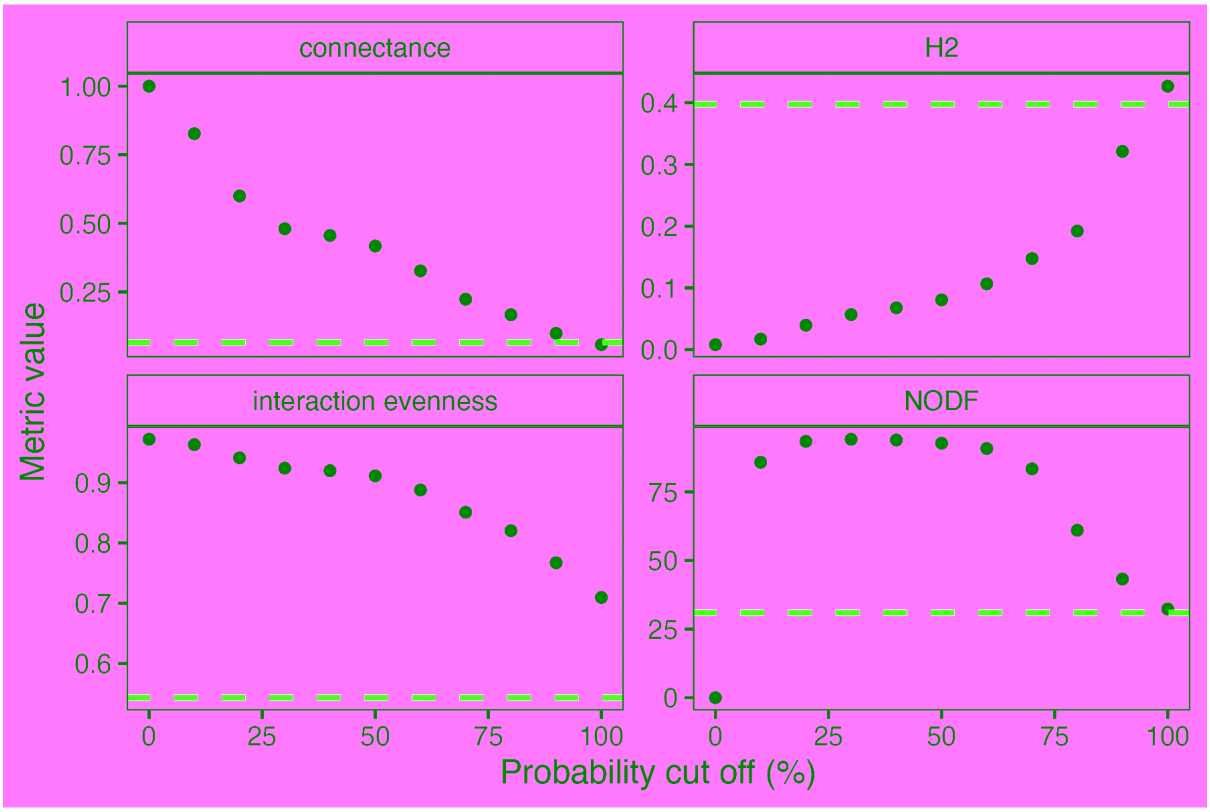

Appendix S1: Figure S2. Calculated network measure based on varying exclusion of modeled interactions at every 10% (i.e., 0, 10, 20, 30, and so forth), or every 0.10 increment of the calculated $z_{i,j}$ occupancy model value. The tested measure value is represented on the y-axis and the x-axis represents how much interaction data is included based on the probability of the interaction. The red dashed line in each subpanel represents the calculated network measure value for the Global Biotic Interactions (GloBI, Poelen et al., 2014) network. The modeled network was consistently more connected, more even, more nested (nestedness metric based on overlap and decreasing fill, NODF), and less specialized (H_2', reciprocal specialization) than the raw GloBI network.

Appendix S1: Table S2. List of sources from the Global Biotic Interactions (GloBI, Poelen et al., 2014) database, the unique number of bee-plant observations, and the total number of bee-plant observations recorded in GloBI. Sources include aggregated data (i.e., data are collected from other sources; e.g., web scraping), collection specimen (e.g., museum or university collection), literature (i.e., primary published literature), or observation (i.e., community science initiatives; e.g., iNaturalist). Sources in bold were retained for the analysis.

Citation	Unique number of bee-plant observations	Total number of bee-plant observations
A. Thessen. 2014. Species associations extracted from EOL text data objects via text mining.	19	19
American Museum of Natural History Invertebrate Zoology Collection	50	166
Archbold Biological Station Arthropod Collection	1	1
Arizona State University	1	1
Bee Biology and Systematics Laboratory	99	750
BLM Mother Lode Field Office: The Bees of Pine Hill Preserve	4	5
C.P. Gillette Museum of Arthropod Diversity	5	42
Carril OM, Griswold T, Haefner J, Wilson JS. (2018) Wild bees of Grand Staircase-Escalante National Monument: richness, abundance, and spatio-temporal beta-diversity. PeerJ 6:e5867 https://doi.org/10.7717/peerj.5867	83	222
Connecticut Agricultural Experiment Station Arthropod Collection	3	4
Cornell University Insect Collection	6	23
Dartmouth College Insect Collection	2	2
Essig Museum of Entomology	15	82
Florida State Collection of Arthropods	1	1
Giselle Muschett & Francisco E. Fontúrbel. 2021. A comprehensive catalogue of plant-pollinator interactions for Chile	1	1
**Guzman, Laura Melissa; Kelly, Tyler; Elle, Elizabeth, 2022, ""A dataset for pollinator diversity and their interactions with plants in the Pacific NorthWest"", https://doi.org/10.5683/SP3/WTEZNH, Borealis, V1**	**135**	**586**
https://mangal.io - the ecological interaction database.	3	3
**iNaturalist Research-grade Observations**	**492**	**2237**
IPBES. (2016). The assessment report of the Intergovernmental Science-Policy Platform on Biodiversity and Ecosystem Services on pollinators, pollination and food production. Table 2.4.3 p88 Zenodo. https://doi.org/10.5281/zenodo.3402857	1	1

**Kenneth S. Norris Center for Natural History, University of California Santa Cruz, Insect Collection**	**228**	**814**
KWP Lepidoptera Collection (Arctos)	1	67
LaManna, JA, Burkle, LA, Belote, RT, Myers, JA. Biotic and abiotic drivers of plant-pollinator community assembly across wildfire gradients. J Ecol. 2020; 00: 1-14. https://doi.org/10.1111/1365-2745.13530.	10	14
Mississippi Entomological Museum	4	8
Museum of Comparative Zoology, Harvard University	2	2
Museum of Southwestern Biology	10	36
National Database Plant Pollinators. Center for Plant Conservation at San Diego Zoo Global. Accessed via https://saveplants.org/national-collection/pollinator-search/ on 2020-06-05.	7	7
Natural History Museum of Los Angeles County	2	3
Nick Balfour, Maria Clara Castellanos, Chris Johnson, Dave Goulson, Andrew Philippides. 2023. The Database of Pollinator Interactions (DoPI). Accessed at https://www.sussex.ac.uk/lifesci/ebe/dopi/ on 2023-12-01.	2	2
Ollerton, J., Trunschke, J., Havens, K., Landaverde-González, P., Keller, A., Gilpin, A.M., … Arnold, S. E. J. (2022). Pollinator-flower interactions in gardens during the COVID-19 pandemic lockdown of 2020. *Journal of Pollination Ecology*, *32*, 87–96. https://doi.org/10.26786/1920-7603(2022)695	1	1
Orthoptera DNA Tissue Collection	9	17
Plant–pollinator community assembly across wildfire gradients	8	10
R. M. Bohart Museum of Entomology	9	24
Redhead, J.W.; Coombes, C.F.; Dean, H.J.; Dyer, R.; Oliver, T.H.; Pocock, M.J.O.; Rorke, S.L.; Vanbergen, A.J.; Woodcock, B.A.; Pywell, R.F. (2018). Plant-pollinator interactions database for construction of potential networks. NERC Environmental Information Data Centre. https://doi.org/10.5285/6d8d5cb5-bd54-4da7-903a-15bd4bbd531b	4	4
RL Minckley Insect and Plant Collection	27	323
Robert L. Minckley San Bernardino Valley from the year 2000 to 2011.	34	34
Rocky Mountain Biological Laboratory Insect Collection	2	2
Santa Barbara Museum of Natural History Entomology Collection	5	10
Sarah E Miller. 12/13/2016. Species associations manually extracted from Onstad, D.W. EDWIP: Ecological Database of the World's Insect Pathogens. Champaign, Illinois: Illinois Natural History Survey, [23/11/2016]. http://insectweb.inhs.uiuc.edu/Pathogens/EDWIP.	1	5
**Schwarz, Benjamin et al. (2021). Data from: Temporal scale-dependence of plant-pollinator networks [Dataset]. Dryad. https://doi.org/10.5061/dryad.qz612jmbp**	**142**	**354**

Seltmann, K., Van Wagner, J., Behm, R., Brown, Z., Tan, E., & Liu, K. (2020). BID: A project to share biotic interaction and ecological trait data about bees (Hymenoptera: Anthophila). UC Santa Barbara: Cheadle Center for Biodiversity and Ecological Restoration. Retrieved from https://escholarship.org/uc/item/1g21k7bf	44	98
**Symbiota Collections of Arthropods Network (SCAN)**	**215**	**3767**
The Purdue Entomological Research Collection	7	227
United States Geological Survey (USGS) Pollinator Library. https://www.npwrc.usgs.gov/pollinator.	3	3
United States National Museum, Entomology Collections	2	2
University of California Santa Barbara Invertebrate Zoology Collection	49	101
University of Colorado Museum of Natural History Entomology Collection	13	29
University of Connecticut Museum	9	52
**University of Kansas Natural History Museum Entomology Division**	**103**	**294**
University of Kentucky Insect Collection	1	1
University of Michigan Museum of Zoology, Division of Insects	3	5
Web of Life. http://www.web-of-life.es.	3	3

Appendix S1: Table S3. Calculated network measures for the raw Global Biotic Interactions (GloBI, Poelen et al., 2014) data and modeled networks. The values for the modeled network here include interactions with a 50% probability or higher. We also include if this value was significantly different relative to the null network values (n = 500), as indicated in the p-value column. We did not include values of connectance in the comparison with null networks as null networks were generated using the same value of connectance as the observed (i.e., GloBI and modeled) networks.

**Metric**	**Network**	**Value**	**p-value** (relative to null networks)
Connectance (proportion of possible links observed in a network, 0-1)	GloBI	0.066	--
	Model	0.417	--
H_2' (reciprocal specialization, 0-1)	GloBI	0.398	$p < 0.001$
	Model	0.081	0.89
Interaction evenness (Shannon's evenness, 0-1)	GloBI	0.543	$p < 0.001$
	Model	0.912	$p < 0.001$
NODF (nestedness metric based on overlap and decreasing fill, 0-100 where 0 is low nestedness)	GloBI	30.955	$p < 0.001$
	Model	92.704	$p < 0.001$

References:

Ascher, J. S., & Pickering, J. (2020). *Discover Life bee species guide and world checklist (Hymenoptera: Apoidea: Anthophila)*. https://www.discoverlife.org/mp/20q?guide=Apoidea_species

Bartomeus, I. (2013). Understanding linkage rules in plant-pollinator networks by using hierarchical models that incorporate pollinator detectability and plant traits. *PLoS ONE*, *8*(7), e69200. https://doi.org/10.1371/journal.pone.0069200

Brady, S. G., Sipes, S., Pearson, A., & Danforth, B. N. (2006). Recent and simultaneous origins of eusociality in halictid bees. *Proceedings of the Royal Society B: Biological Sciences*, *273*(1594), 1643–1649. https://doi.org/10.1098/rspb.2006.3496

Bosch, J., Retana, J., & Cerdá, X. (1997). Flowering phenology, floral traits and pollinator composition in a herbaceous Mediterranean plant community. *Oecologia*, *109*(4), 583–591. https://doi.org/10.1007/s004420050120

Danforth, B. N., Minckley, R. L., Neff, J. L., & Fawcett, F. (2019). *The solitary bees: Biology, evolution, conservation*. Princeton University Press.

Jepson Flora Project (eds.). (2022). Jepson eFlora. https://ucjeps.berkeley.edu/eflora/

Lee, M.J., Diao, C. & Seltmann, K.C. (2025). lee-michellej/globi_msomAndsciChecklists: Manuscript Release (Data) (v5). Zenodo. https://doi.org/10.5281/zenodo.13539345

Olesen, J. M., Dupont, Y. L., Ehlers, B. K., & Hansen, D. M. (2007). The openness of a flower and its number of flower-visitor species. *TAXON*, *56*(3), 729–736. https://doi.org/10.2307/25065856

Ostwald, M. M., Gonzalez, V. H., Chang, C., Vitale, N., Lucia, M., & Seltmann, K. C. (2024). Toward a functional trait approach to bee ecology. *Ecology and Evolution*, *14*(10), e70465. https://doi.org/10.1002/ece3.70465

Poelen, J. H., Simons, J. D., & Mungall, C. J. (2014). Global biotic interactions: An open infrastructure to share and analyze species-interaction datasets. *Ecological Informatics*, *24*, 148–159. https://doi.org/10.1016/j.ecoinf.2014.08.005

Seltmann, K., Allen, J., Brown, B. V., Carper, A., Engel, M. S., Franz, N., Gilbert, E., Grinter, C., Gonzalez, V. H., Horsley, P., Lee, S., Maier, C., Miko, I., Morris, P., Oboyski, P., Pierce, N. E., Poelen, J., Scott, V. L., Smith, M., … Tuker, E. (2021). Announcing Big-Bee: An initiative to promote understanding of bees through image and trait digitization. *Biodiversity Information Science and Standards*, *5*(e74037). https://escholarship.org/uc/item/0937b5gp

Soucy, S. L. (2002). Nesting Biology and Socially Polymorphic Behavior of the Sweat Bee *Halictus rubicundus* (Hymenoptera: Halictidae). *Annals of the Entomological Society of America*, *95*(1), 57–65. https://doi.org/10.1603/0013-8746(2002)095[0057:NBASPB]2.0.CO;2

Wilson, J. S., & Carril, O. M. (2015). The Bees in Your Backyard: A Guide to North America's Bees. In *The Bees in Your Backyard*. Princeton University Press. https://doi.org/10.1515/9781400874156
