# Supplementary material for: Leveraging local species data, a global database, and an occupancy model to explore bee–plant interactions
Source: Ecol Appl. 2026 Mar 24;36(2):e70221. doi: 10.1002/eap.70221 (PMC13012871; doi:10.1002/eap.70221)
Supplement: Supplementary file 2 — Appendix S2. [file EAP-36-e70221-s002.pdf]

Appendix S2

Leveraging local species data, a global database, and an occupancy model to explore bee–plant interactions

Michelle J. Lee, Graziella V. DiRenzo, Chengyi Diao, Katja C. Seltmann

Ecological Applications

Any use of trade, firm, or product names is for descriptive purposes only and does not imply endorsement by the U.S. Government.

Appendix S2: Methods

Occupancy model - To explore broad phylogenetic constraints on bee-plant interactions, such as identifying families with low interaction probabilities (potentially "forbidden interactions"), we ran five additional models with random effects at the following levels:

- Bee family
- Plant species
- Plant family
- Bee and plant family
- No bee or plant family or species

To build these additional five models, we used the model written in the main manuscript as the base, where all parameters and covariates were kept consistent, and modified the random effects structure in the following ways:

Bee Family Random Effect Model

- Ecological Model:
  - **Appendix S2: Equation S1** $\text{logit}(\Psi_{i,j}) = \beta 1 + \beta 2 \cdot \textit{Bee.size}_i + \beta 3 \cdot \textit{Bee.solitary}_i + \beta 4 \cdot \textit{Other.flower.color}_j + \beta 5 \cdot \textit{Blue.flower.color}_j + \beta 6 \cdot \textit{White.flower.color}_j + \beta 7 \cdot \textit{Flower.shape}_j + u_{\textit{Bee family(i)}}$
  - **Appendix S2: Equation S2** $u_{\textit{Bee family(i)}} \sim \text{Normal}(0, \sigma_\Psi^2)$
- Detection Model:
  - **Appendix S2: Equation S3** $\text{logit}(p_{i,j,k}) = \delta 1 + \delta 2 \cdot \textit{Bee.strip}_i + \delta 3 \cdot \textit{Bee.size}_i + \delta 4 \cdot \textit{Lit.citation.type}_k + \delta 5 \cdot \textit{Col.citation.type}_k + \delta 6 \cdot \textit{Agg.citation.type}_k + \delta 7 \cdot \textit{Other.flower.color}_j + \delta 8 \cdot \textit{Blue.flower.color}_j + \delta 9 \cdot \textit{White.flower.color}_j + \delta_{10} \cdot \textit{Flower.shape}_j + v_{\textit{Bee family(i)}}$
  - **Appendix S2: Equation S4** $v_{\textit{Bee family(i)}} \sim \text{Normal}(0, \sigma_p^2)$

Plant Species Random Effect Model

- Ecological Model:
  - **Appendix S2: Equation S5** $\text{logit}(\Psi_{i,j}) = \beta 1 + \beta 2 \cdot \textit{Bee.size}_i + \beta 3 \cdot \textit{Bee.solitary}_i + \beta 4 \cdot \textit{Other.flower.color}_j + \beta 5 \cdot \textit{Blue.flower.color}_j + \beta 6 \cdot \textit{White.flower.color}_j + \beta 7 \cdot \textit{Flower.shape}_j + u_{\textit{Plant species(i)}}$
  - **Appendix S2: Equation S6** $u_{\textit{Plant species(i)}} \sim \text{Normal}(0, \sigma_\Psi^2)$
- Detection Model:
  - **Appendix S2: Equation S7** $\text{logit}(p_{i,j,k}) = \delta 1 + \delta 2 \cdot \textit{Bee.strip}_i + \delta 3 \cdot \textit{Bee.size}_i + \delta 4 \cdot \textit{Lit.citation.type}_k + \delta 5 \cdot \textit{Col.citation.type}_k + \delta 6 \cdot \textit{Agg.citation.type}_k + \delta 7 \cdot \textit{Other.flower.color}_j + \delta 8 \cdot \textit{Blue.flower.color}_j + \delta 9 \cdot \textit{White.flower.color}_j + \delta_{10} \cdot \textit{Flower.shape}_j + v_{\textit{Plant species(i)}}$
  - **Appendix S2: Equation S8** $v_{\textit{Plant species(i)}} \sim \text{Normal}(0, \sigma_p^2)$

Plant Family Random Effect Model

- Ecological Model:
  - **Appendix S2: Equation S9** $\text{logit}(\Psi_{i,j}) = \beta 1 + \beta 2 \cdot \textit{Bee.size}_i + \beta 3 \cdot \textit{Bee.solitary}_i + \beta 4 \cdot \textit{Other.flower.color}_j + \beta 5 \cdot \textit{Blue.flower.color}_j + \beta 6 \cdot \textit{White.flower.color}_j + \beta 7 \cdot \textit{Flower.shape}_j + u_{\textit{Plant family(i)}}$
  - **Appendix S2: Equation S10** $u_{\textit{Plant family(i)}} \sim \text{Normal}(0, \sigma_{\Psi}^2)$

- Detection Model:
  - **Appendix S2: Equation S11** $\text{logit}(p_{i,j,k}) = \delta 1 + \delta 2 \cdot \textit{Bee.strip}_i + \delta 3 \cdot \textit{Bee.size}_i + \delta 4 \cdot \textit{Lit.citation.type}_k + \delta 5 \cdot \textit{Col.citation.type}_k + \delta 6 \cdot \textit{Agg.citation.type}_k + \delta 7 \cdot \textit{Other.flower.color}_j + \delta 8 \cdot \textit{Blue.flower.color}_j + \delta 9 \cdot \textit{White.flower.color}_j + \delta_{10} \cdot \textit{Flower.shape}_j + v_{\textit{Plant family(i)}}$
  - **Appendix S2: Equation S12** $v_{\textit{Plant family(i)}} \sim \text{Normal}(0, \sigma_{\text{p}}^2)$

Bee and Plant Family Random Effect Model

- Ecological Model:
  - **Appendix S2: Equation S13** $\text{logit}(\Psi_{i,j}) = \beta 1 + \beta 2 \cdot \textit{Bee.size}_i + \beta 3 \cdot \textit{Bee.solitary}_i + \beta 4 \cdot \textit{Other.flower.color}_j + \beta 5 \cdot \textit{Blue.flower.color}_j + \beta 6 \cdot \textit{White.flower.color}_j + \beta 7 \cdot \textit{Flower.shape}_j + u_{\textit{Plant family(i)}} + g_{\textit{Bee family(i)}}$
  - **Appendix S2: Equation S14** $u_{\textit{Plant family(i)}} \sim \text{Normal}(0, \sigma_{\Psi}^2)$
  - **Appendix S2: Equation S15** $g_{\textit{Bee family(i)}} \sim \text{Normal}(0, \sigma_{\Psi}^2)$
- Detection Model:
  - **Appendix S2: Equation S16** $\text{logit}(p_{i,j,k}) = \delta 1 + \delta 2 \cdot \textit{Bee.strip}_i + \delta 3 \cdot \textit{Bee.size}_i + \delta 4 \cdot \textit{Lit.citation.type}_k + \delta 5 \cdot \textit{Col.citation.type}_k + \delta 6 \cdot \textit{Agg.citation.type}_k + \delta 7 \cdot \textit{Other.flower.color}_j + \delta 8 \cdot \textit{Blue.flower.color}_j + \delta 9 \cdot \textit{White.flower.color}_j + \delta_{10} \cdot \textit{Flower.shape}_j + v_{\textit{Plant family(i)}} + d_{\textit{Bee family(i)}}$
  - **Appendix S2: Equation S17** $v_{\textit{Plant family(i)}} \sim \text{Normal}(0, \sigma_{\text{p}}^2)$
  - **Appendix S2: Equation S18** $d_{\textit{Bee family(i)}} \sim \text{Normal}(0, \sigma_{\text{p}}^2)$

No Random Effects Model

- Ecological Model:
  - **Appendix S2: Equation S19** $\text{logit}(\Psi_{i,j}) = \beta 1 + \beta 2 \cdot \textit{Bee.size}_i + \beta 3 \cdot \textit{Bee.solitary}_i + \beta 4 \cdot \textit{Other.flower.color}_j + \beta 5 \cdot \textit{Blue.flower.color}_j + \beta 6 \cdot \textit{White.flower.color}_j + \beta 7 \cdot \textit{Flower.shape}_j$
- Detection Model:
  - **Appendix S2: Equation S20** $\text{logit}(p_{i,j,k}) = \delta 1 + \delta 2 \cdot \textit{Bee.strip}_i + \delta 3 \cdot \textit{Bee.size}_i + \delta 4 \cdot \textit{Lit.citation.type}_k + \delta 5 \cdot \textit{Col.citation.type}_k + \delta 6 \cdot \textit{Agg.citation.type}_k + \delta 7 \cdot \textit{Other.flower.color}_j + \delta 8 \cdot \textit{Blue.flower.color}_j + \delta 9 \cdot \textit{White.flower.color}_j + \delta_{10} \cdot \textit{Flower.shape}_j$

Family-level interaction probability - To calculate the family-level interaction probability for the "Bee Family Random Effects Model" and the "Plant Family Random Effects Model", we averaged ψ values across all species that belong to the same family.

Parameter comparisons- To quantify differences among parameter estimates, we computed the proportion of iterations where one parameter (e.g., *a*) was greater than another parameter (e.g., *b*), following Ruiz-Gutiérrez et al., (2010). This is analogous to calculating the probability that parameter *a* is greater than parameter *b* [written as Pr($a > b$)] and can be directly interpreted as the probability that the first parameter is greater than the second. Extreme values (close to 0 and 1) suggest that the parameters are significantly different, whereas values near 0.50 suggest that estimates are similar.

Model fitting- We analyzed all of the models using a Bayesian approach in programs R (R Core Team 2023) and nimble (package *nimble*; de Valpine et al., 2017, 2022). The models were run on the U.S. Geological Survey (USGS) Hovenweep supercomputer, accessed through the USGS Advanced Research Computing facility (Falgout et al. 2025). We ran the models each for 250,000 iterations with a burn-in of 50,000 iterations and thinning by 10. We ran each model with a total of three chains.

We ran each model type with six combinations of priors, where covariate parameters had Normal(mean = 0, standard deviation = 1, 3, or 5) and variance parameters = Normal(mean = 0, standard deviation = 2 or 3). Below, we only report one converged model in the text. If multiple models converged for a single model type, then we used the model run with the priors that had the largest standard deviation. The final models included the following priors for each model:

- Bee family
    - Covariate parameters = Normal(mean = 0, standard deviation = 5)
    - Variance parameters = Normal(mean = 0, standard deviation = 3)
- Plant species
    - Covariate parameters = Normal(mean = 0, standard deviation = 5)
    - Variance parameters = Normal(mean = 0, standard deviation = 3)
- Plant family
    - Covariate parameters = Normal(mean = 0, standard deviation = 5)
    - Variance parameters = Normal(mean = 0, standard deviation = 3)
- Bee and plant family
    - Covariate parameters = Normal(mean = 0, standard deviation = 3)
    - Variance parameters = Normal(mean = 0, standard deviation = 3)
- No bee or plant family or species
    - Covariate parameters = Normal(mean = 0, standard deviation = 5)
    - Variance parameters = Normal(mean = 0, standard deviation = 3)

All variance parameters were truncated at 0 with no upper limit. We assessed convergence using the $\hat{R}$ statistic (Brooks and Gelman 1998) and visually inspected traceplots. We did not conduct a goodness of fit test because goodness of fit tests may indicate poor fit in cases with small sample size (DiRenzo et al. 2023).

Appendix S2: Results

Ecological results - We found mixed results related to how the probability of interacting with a plant relates to bee size, where the only significantly negative relationship was detected for the bee-species random effect model, a trend towards significance was detected for the 'bee and plant family random effects model', and the remaining four models showed non-significant slopes (Appendix S2: Figure S1).

In terms of bee sociality and bee-plant interaction probability, we found that 5 out of 6 models detected that solitary bees have a higher probability of interacting with a flower than social bees (Appendix S2: Figure S2).

In terms of flower color and bee-plant interaction probability, we found that 5 out of 6 models found that blue flowers are visited more frequently than yellow flowers (Appendix S2: Figure S3). One out of 6 models found that yellow flowers are visited more frequently than white flowers (bee and plant family model), and one out of 6 models found that other colored flowers are visited more frequently than yellow flowers (bee family model). All other comparisons were not significant (Appendix S2: Figure S3).

In terms of flower shape and bee-plant interaction probability, we found that 3 out of 6 models found that not bowl flowers are visited more frequently than bowl flowers (bee species, bee family, and bee and plant family models; Appendix S2: Figure S4).

Detection results - In terms of bee stripes and bee-plant detection probability, we found that 5 out of 6 models found that not striped bees are detected more frequently on flowers than striped bees (Appendix S2: Figure S5).

In terms of source type and bee-plant detection probability, we found that 6 out of 6 models found that observation source types detect bee-plant interactions more frequently than any other source type (Appendix S2: Figure S6).

In terms of flower color and bee-plant detection probability, we found that 3 out of 6 models found that yellow flowers detect bee-plant interactions more frequently than "other" colored flowers (bee species, bee family, and no bee and plant family models; Appendix S2: Figure S7), 1 out of 6 models found that blue flowers detect bee-plant interactions more frequently than yellow colored flowers (Appendix S2: Figure S7), and 1 out of 6 models found that white flowers detect bee-plant interactions more frequently than yellow colored flowers (Appendix S2: Figure S7).

In terms of flower shape and bee-plant detection probability, we found that 4 out of 6 models found that bee-plant interactions are more frequently detected on bowl flowers than not bowl flowers (Appendix S2: Figure S8).

In terms of bee size and bee-plant detection probability, we found that 2 out of 6 models found that bee size was positively related to the probability of detecting bee-plant interactions (Appendix S2: Figure S9).

Family-level interaction probability - Most bee and plant families had low interaction probabilities (Appendix S2: Figure S10), where 4 of 5 bee families had plant interaction probabilities of less than 0.20 and 68 of 68 plant families had bee interaction probabilities less than 0.20. The one bee family that had an interaction probability greater than 0.20 was Colletidae, which had 5 total species- contributing to the low precision.

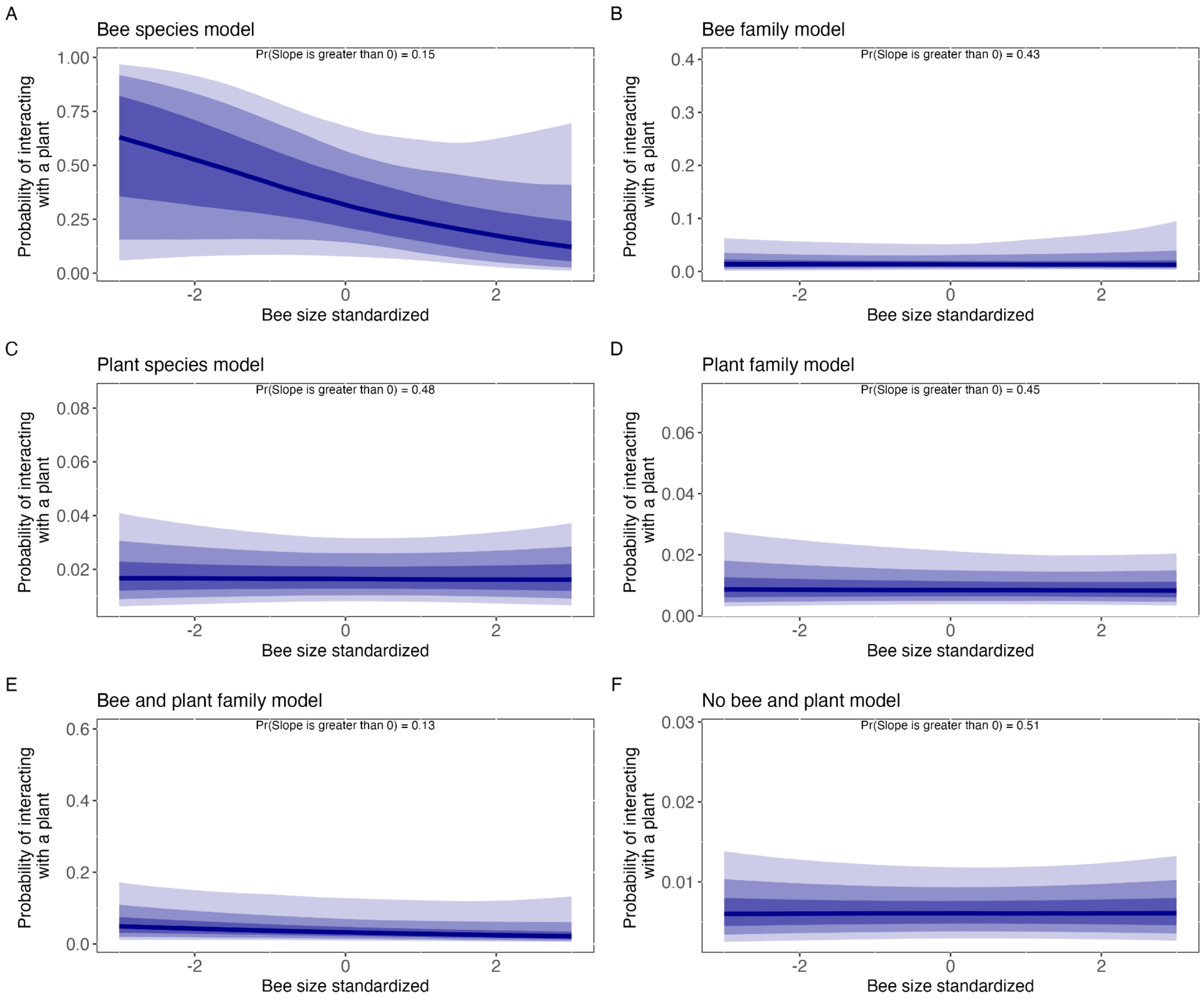

Appendix S2: Figure S1. Probability of interaction versus bee size. Each panel shows a different model as referred to using its random effects structure and labeled in the title of the panel. The shaded regions display the 50%, 80%, and 95% credible intervals (from darkest to lightest shading), derived from the posterior distribution of the Markov chain Monte Carlo (MCMC) samples, with the solid line indicating the posterior mean. The probability the slope estimate is greater than zero is labeled in the top center of each panel.

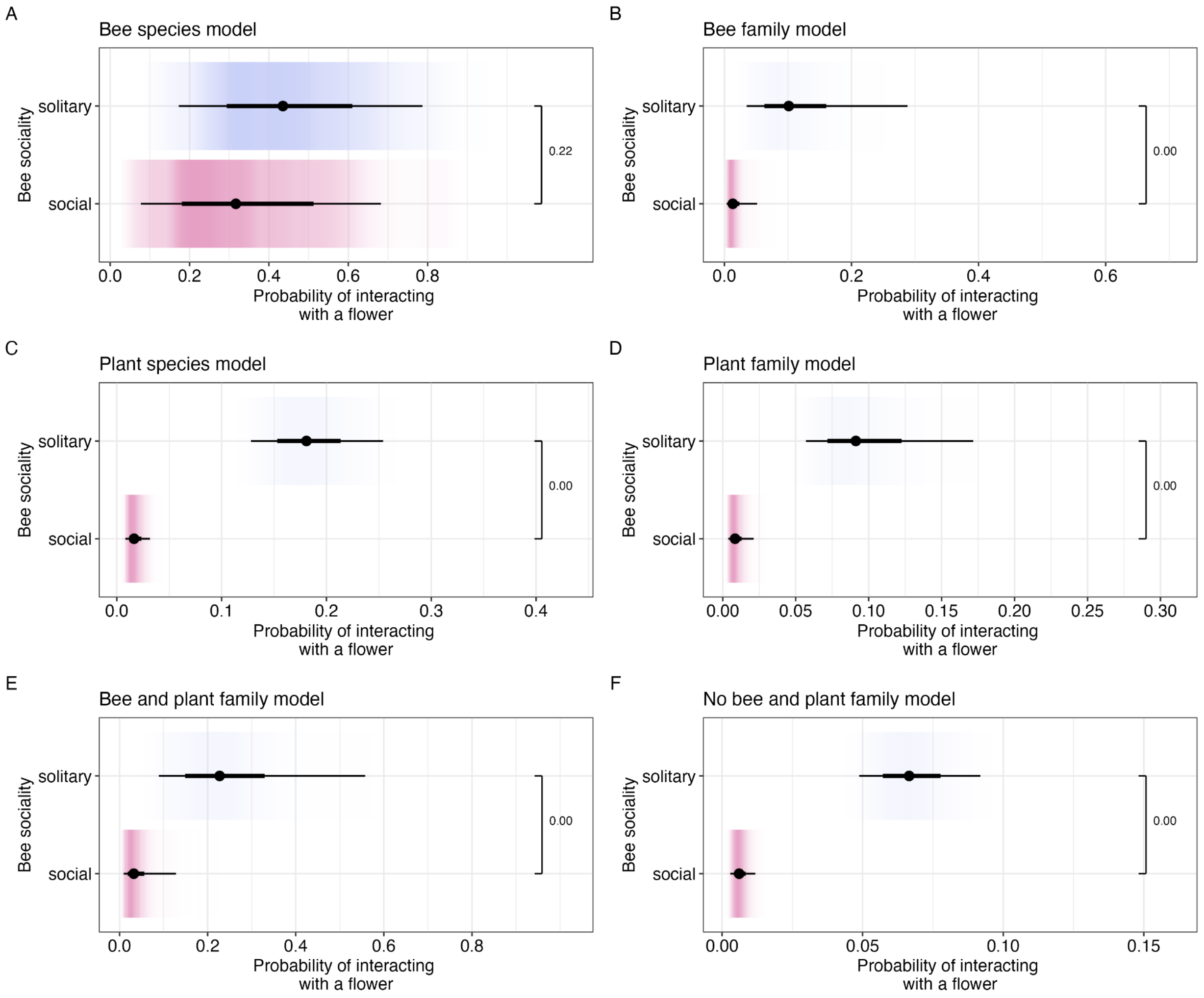

Appendix S2: Figure S2. Probability of interaction versus bee sociality. Each panel shows a different model as referred to using its random effects structure and labeled in the title of the panel. The black dot is the posterior mean and the thick and thin horizontal lines are the 66% and 95 % credible intervals, respectively. The color gradient backgrounds are kernel-smoothed densities of the posterior samples, with darker color indicating where the distribution is most concentrated. The brackets in each panel represent the comparison between social and solitary bees. The value to the right of the bracket is the probability that social bees interact more frequently with flowers than solitary bees.

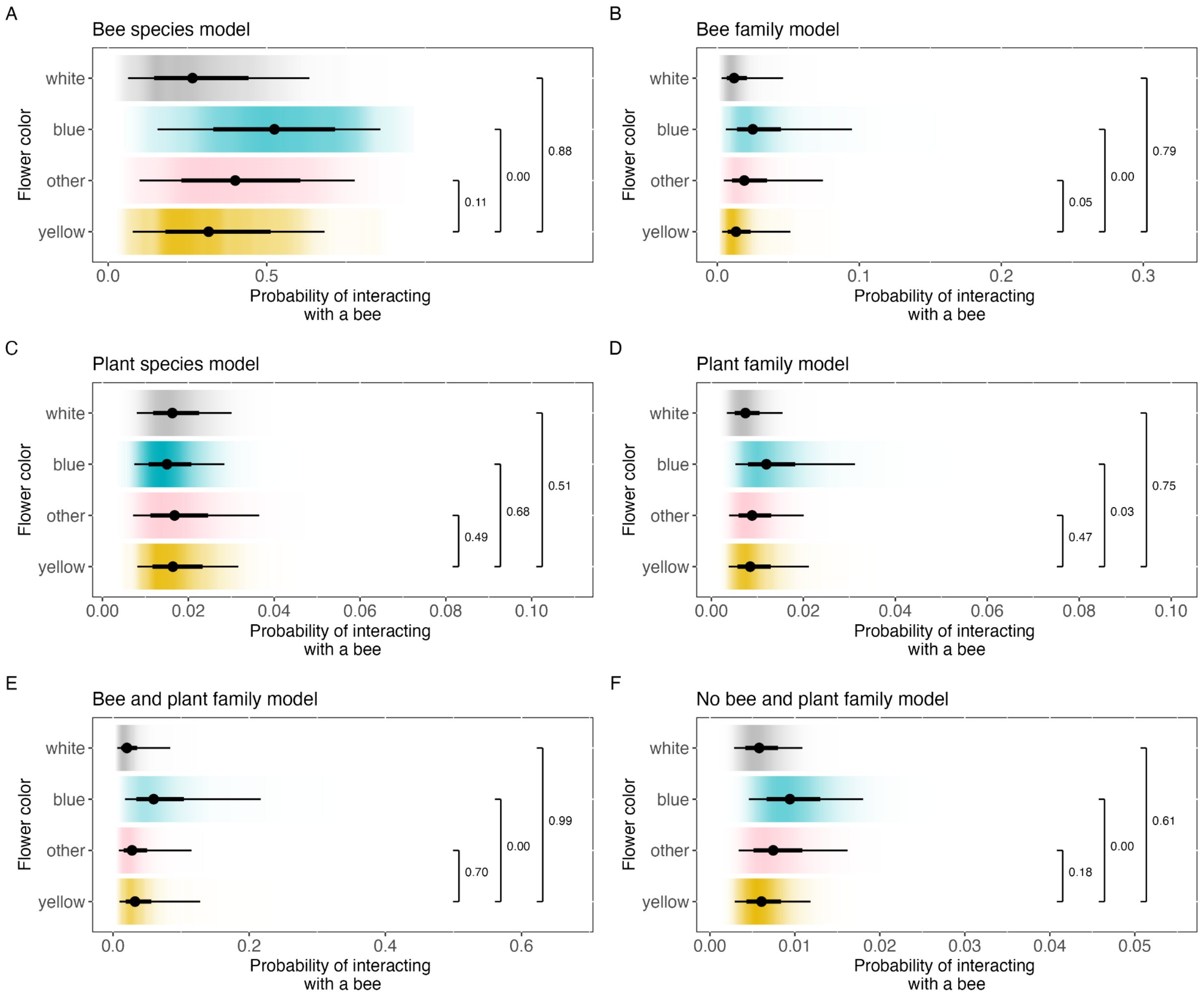

Appendix S2: Figure S3. Probability of interaction versus flower color. Each panel shows a different model as referred to using its random effects structure and labeled in the title of the panel. The black dot is the posterior mean and the thick and thin horizontal lines are the 66% and 95 % credible intervals, respectively. The color gradient backgrounds are kernel-smoothed densities of the posterior samples, with darker color indicating where the distribution is most concentrated. The brackets in each panel represent several comparisons between yellow and several other flower colors. The value to the right of the bracket is the probability that yellow flowers interact with bees more frequently than the flower color it is linked to.

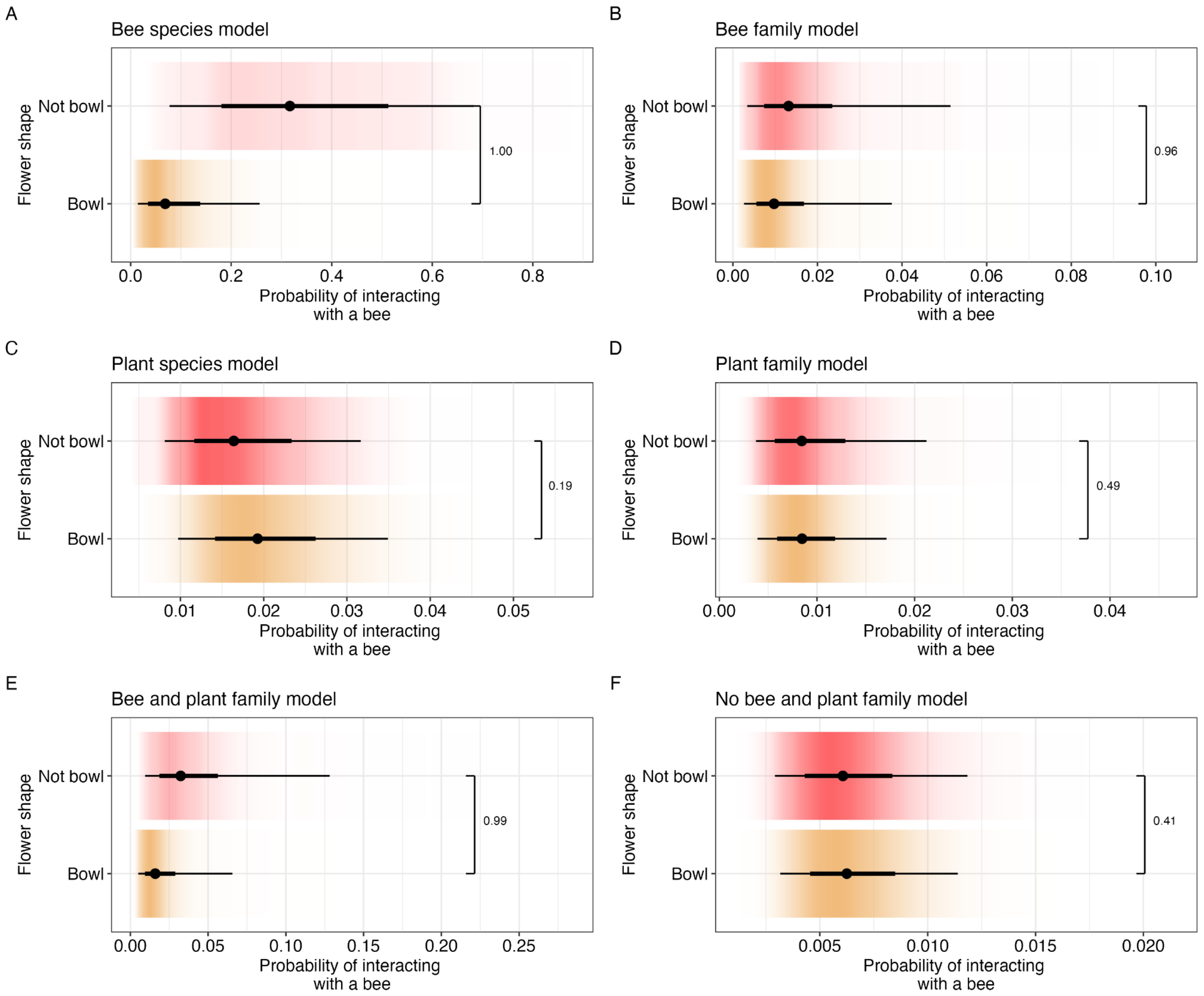

Appendix S2: Figure S4. Probability of interaction versus flower shape. Each panel shows a different model as referred to using its random effects structure and labeled in the title of the panel. The black dot is the posterior mean and the thick and thin horizontal lines are the 66% and 95 % credible intervals, respectively. The color gradient backgrounds are kernel-smoothed densities of the posterior samples, with darker color indicating where the distribution is most concentrated. The brackets in each panel represent the comparison between bowl and not bowl flower shapes. The value to the right of the bracket is the probability that bowl shaped flowers interact with bees more frequently than not bowl shaped flowers.

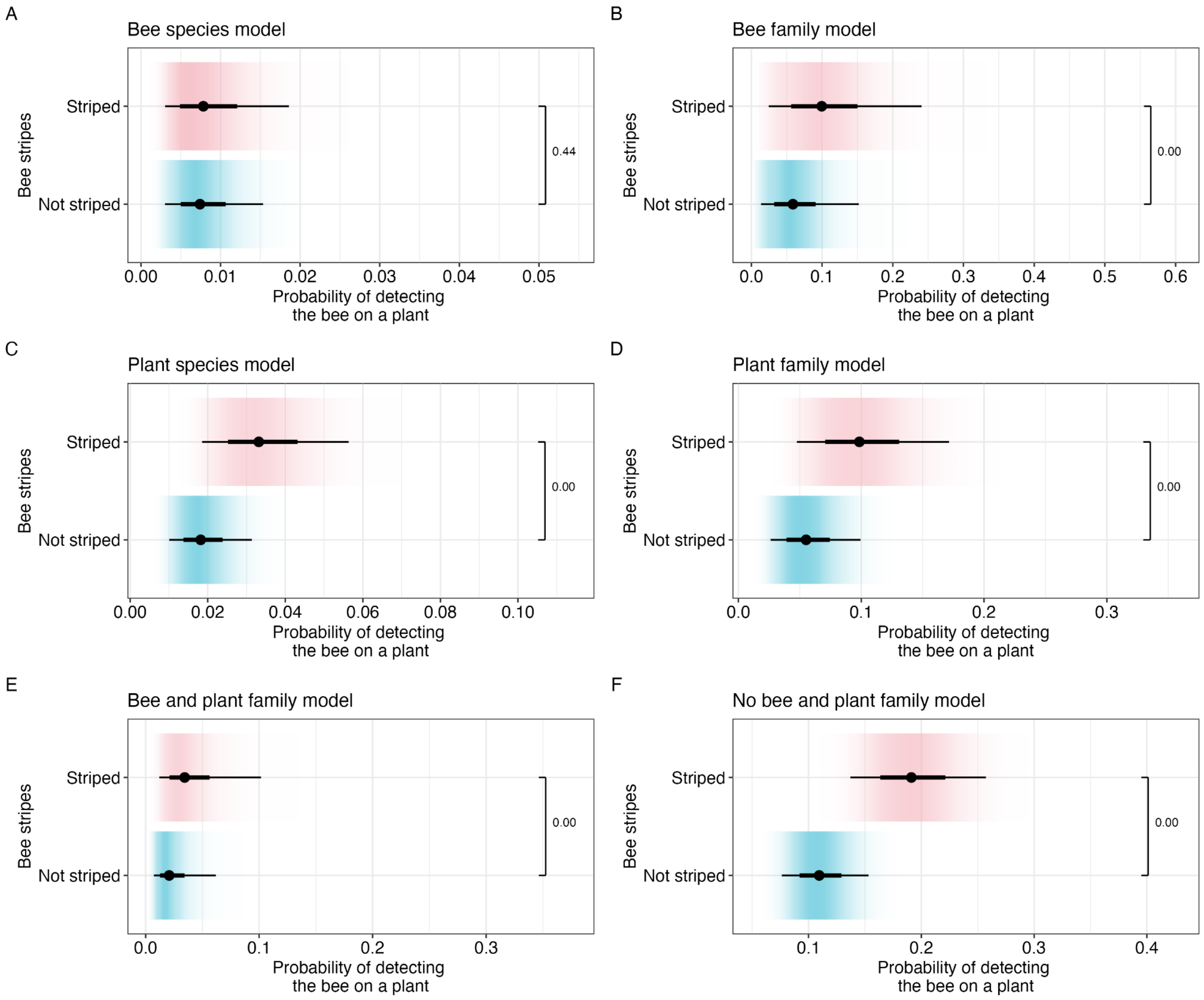

Appendix S2: Figure S5. Probability of interaction versus bee stripes. Each panel shows a different model as referred to using its random effects structure and labeled in the title of the panel. The black dot is the posterior mean and the thick and thin horizontal lines are the 66% and 95 % credible intervals, respectively. The color gradient backgrounds are kernel-smoothed densities of the posterior samples, with darker color indicating where the distribution is most concentrated. The brackets in each panel represent the comparison between not striped and striped bees. The value to the right of the bracket is the probability that not striped bees are detected on flowers more frequently than striped bees.

Appendix

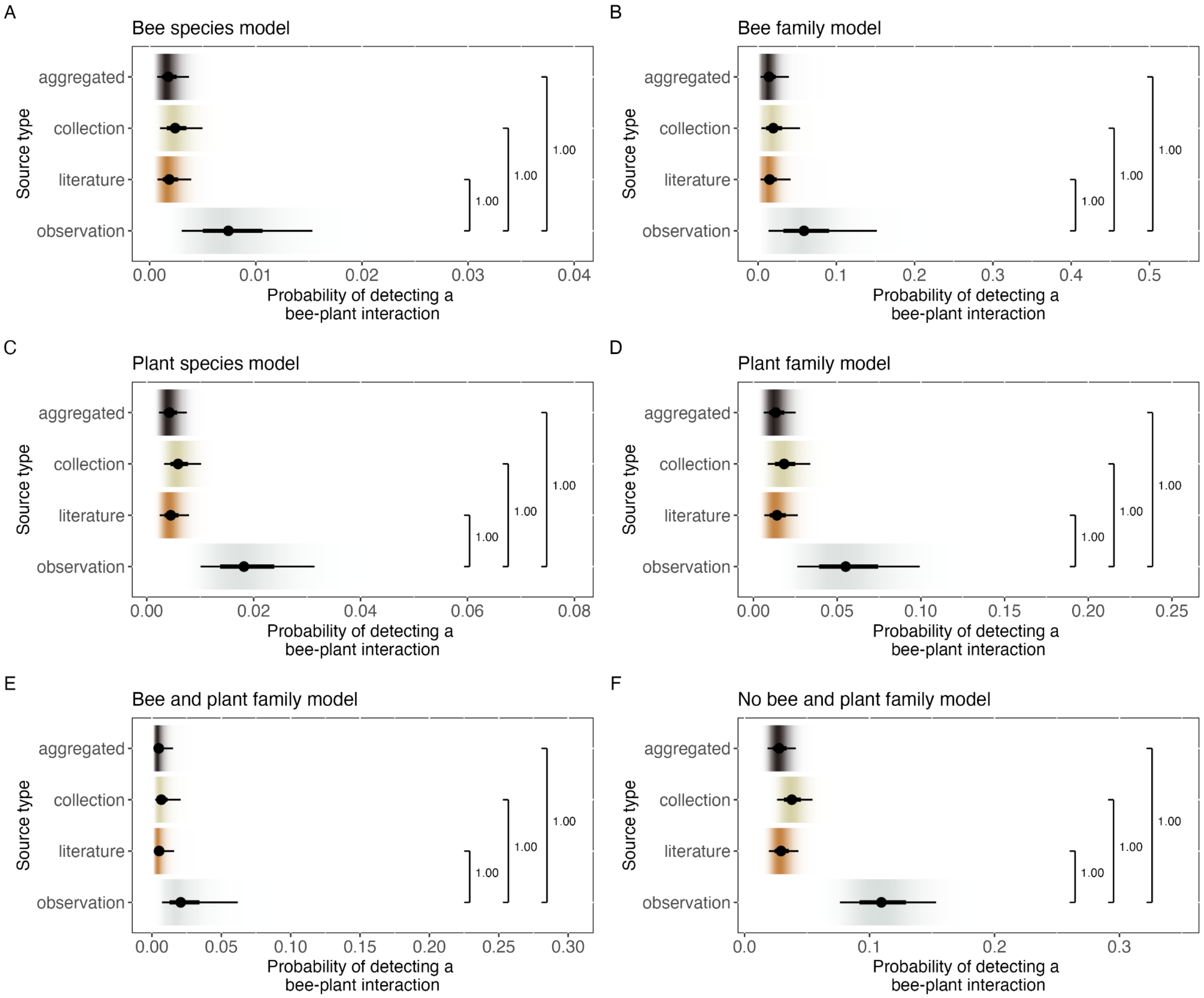

S2: Figure S6. Probability of interaction versus source type. Each panel shows a different model as referred to using its random effects structure and labeled in the title of the panel. The black dot is the posterior mean and the thick and thin horizontal lines are the 66% and 95 % credible intervals, respectively. The color gradient backgrounds are kernel-smoothed densities of the posterior samples, with darker color indicating where the distribution is most concentrated. The brackets in each panel represent several comparisons between the observation source type and several other source types. The value to the right of the bracket is the probability that observation source types detect bee-plant interactions more frequently than the source type it is linked to.

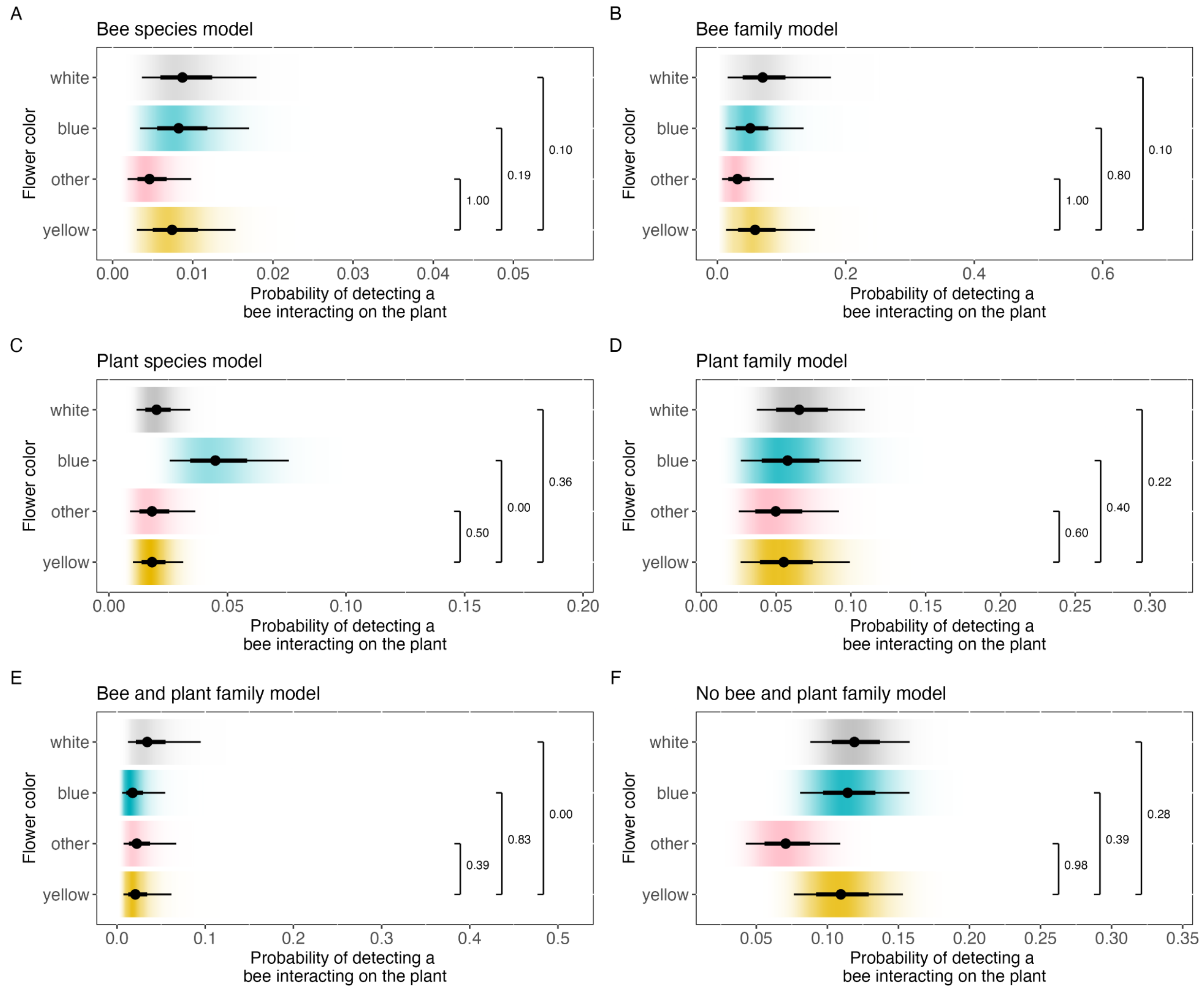

Appendix S2: Figure S7. Probability of interaction versus flower color. Each panel shows a different model as referred to using its random effects structure and labeled in the title of the panel. The black dot is the posterior mean and the thick and thin horizontal lines are the 66% and 95 % credible intervals, respectively. The color gradient backgrounds are kernel-smoothed densities of the posterior samples, with darker color indicating where the distribution is most concentrated. The brackets in each panel represent several comparisons between yellow flowers and several other flower colors. The value to the right of the bracket is the probability that yellow flowers have bee-plant interactions detected more frequently than the flower color that it is linked to.

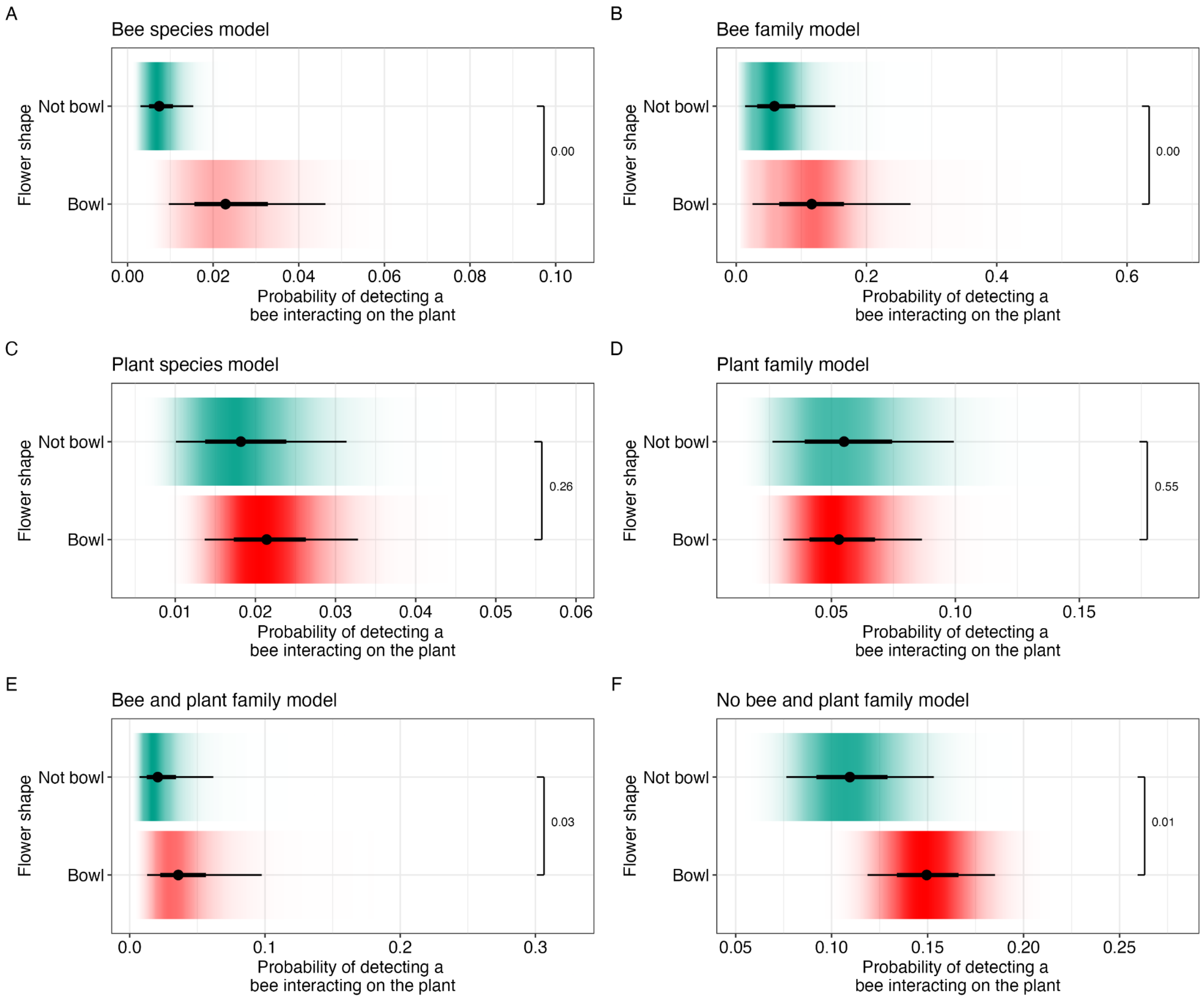

Appendix S2: Figure S8. Probability of interaction versus flower shape. Each panel shows a different model as referred to using its random effects structure and labeled in the title of the panel. The black dot is the posterior mean and the thick and thin horizontal lines are the 66% and 95 % credible intervals, respectively. The color gradient backgrounds are kernel-smoothed densities of the posterior samples, with darker color indicating where the distribution is most concentrated. The brackets in each panel represent the comparison between bowl and not bowl flower shapes. The value to the right of the bracket is the probability that bowl shaped flowers have a bee detected on them more frequently than not bowl shaped flowers.

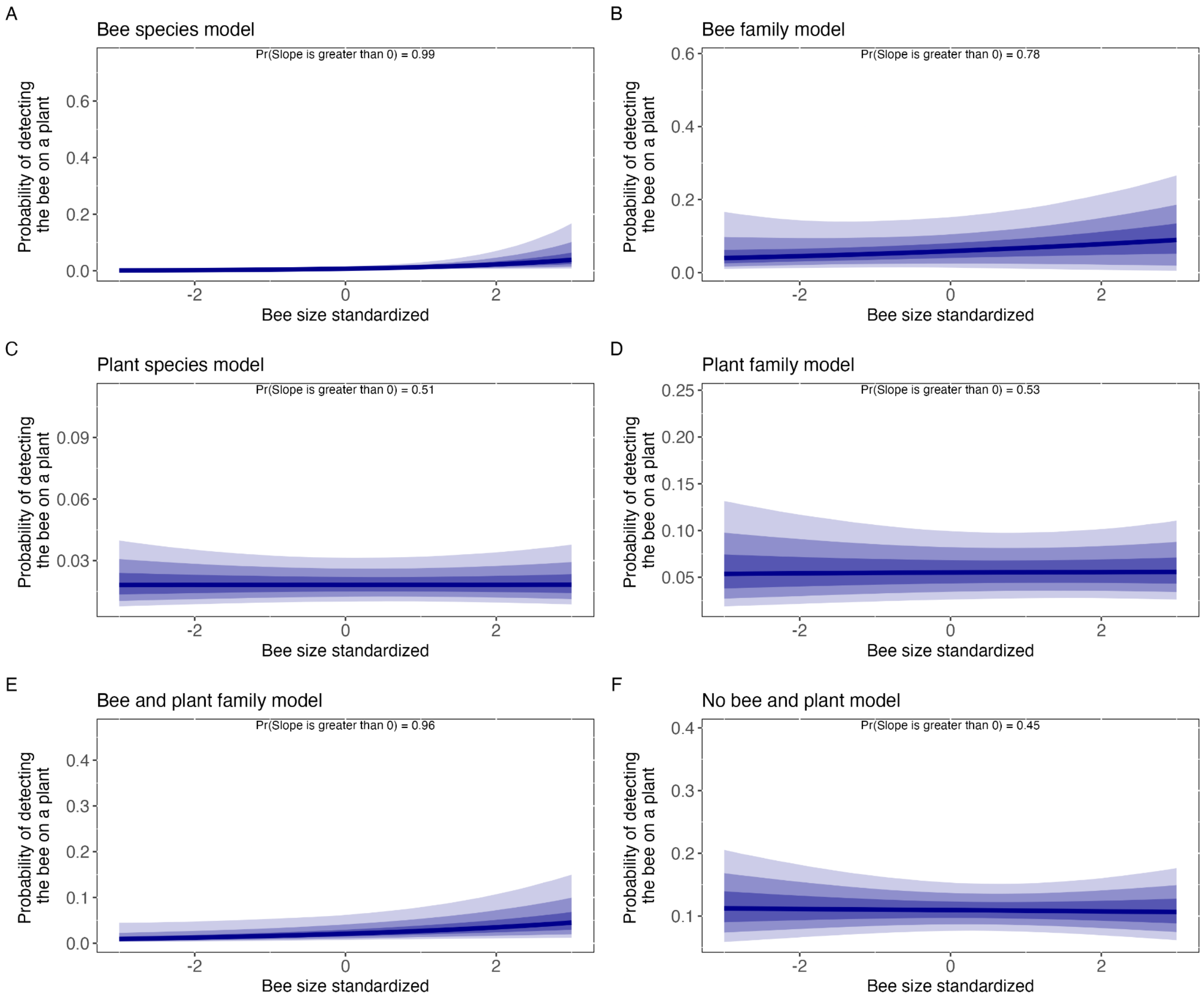

Appendix S2: Figure S9. Probability of interaction versus bee size. Each panel shows a different model as referred to using its random effects structure and labeled in the title of the panel. The shaded regions display the 50%, 80%, and 95% credible intervals (from darkest to lightest shading), derived from the posterior distribution of the Markov chain Monte Carlo (MCMC) samples, with the solid line indicating the posterior mean. The probability the slope estimate is greater than zero is labeled in the top center of each panel.

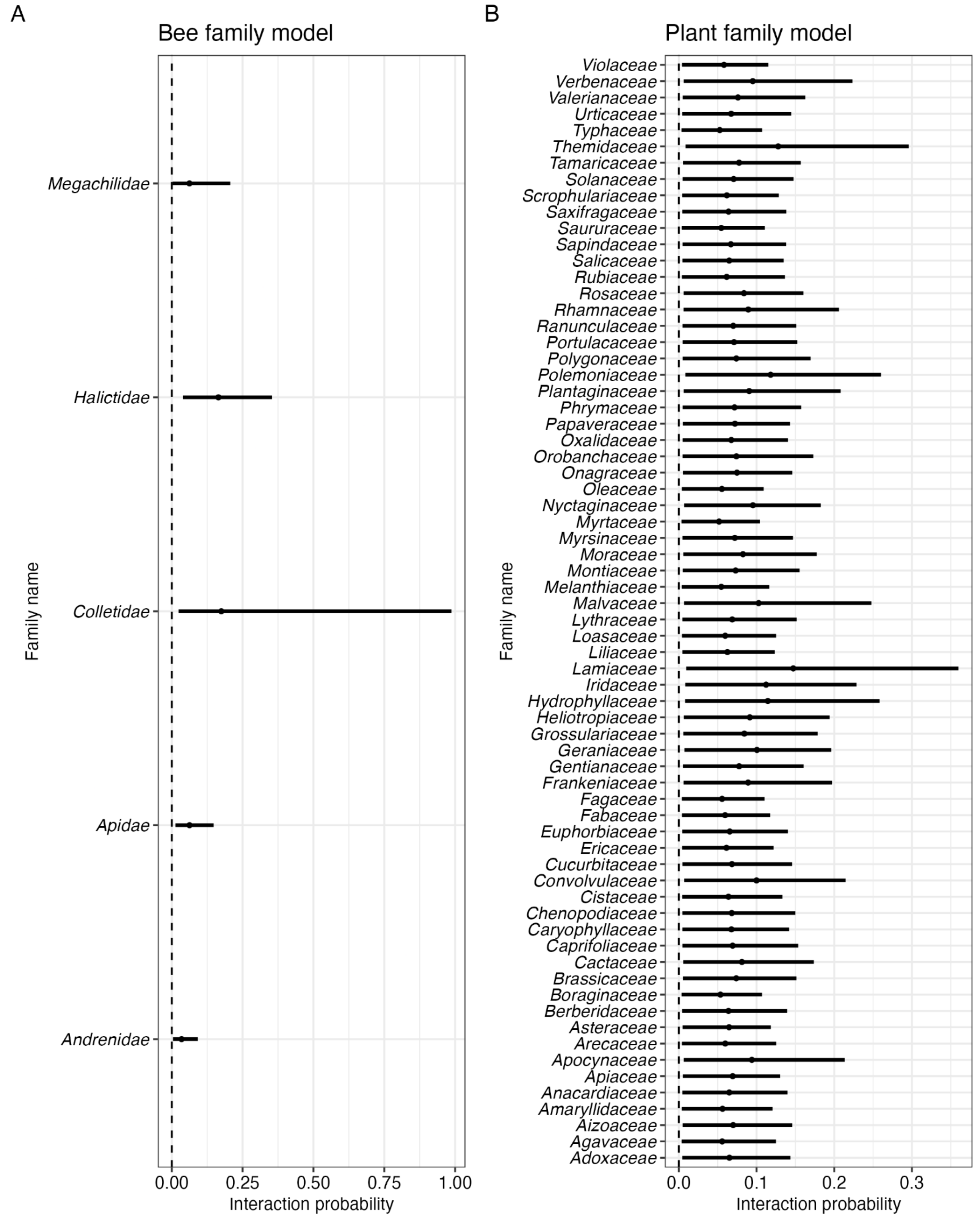

Appendix S2: Figure S10. Probability of interaction with flowers by bee family (panel A) and probability of interaction with bees by plant family (panel B) for the bee family random effects model and plant family random effects model, respectively. The black points represent the mean

and the point ranges are the 95% credible intervals estimated by the corresponding occupancy models.

Appendix S2: Discussion

Why are we detecting different results across the six models?

Changing the random-effects structure changes where the model attributed the unexplained variation. For example, when we include a random intercept for plant species, much of the plant-specific signal (color, reward, phenology, etc.) is absorbed in the random intercept, so a fixed effect such as flower color appears weaker—even though the underlying biology has not changed. In contrast, the no random effects model forces all residual variance into the fixed effects terms, inflating their apparent importance.

The results across all six models illustrate this dynamic clearly:

- Bee sociality impacts the probability a bee interacts with a plant, where solitary bees have a higher interaction probability than social bees (Appendix S2: Figure S2).

- Bee stripes (detection) is significant in every model except the bee-species formulation (Appendix S2: Figure S5).

- Bee size (ecological process) shows a negative slope only when the species intercepts are present (Appendix S2: Figure S1-A) and is effectively zero once plant or family effects are added, indicating that bee size covaries with other taxonomic attributes that the broader random effects now capture.

In short, each additional taxonomic level in the random-effects hierarchy absorbs a different part of the variance, redistributing what is left for the fixed effects to explain. Divergent effect sizes across models therefore reflect partitioning rules rather than contradictory ecological truths, and the ensemble of models provides a useful sensitivity check on how robust each covariate signal really is.

Why is there low variability in mean interaction probability across bee and plant families?

The family-level interaction probability figure highlights that most bee and plant families have low mean interaction probabilities (<0.20), suggesting that interactions are not evenly distributed across taxonomic groups. Notably, all 68 plant families had mean interaction probabilities below 0.20, as did four out of five bee families. The exception, Colletidae, had a higher estimated interaction probability but also exhibited a wide credible interval, likely due to the small number of species (n = 5) contributing to the estimate. We accounted for bee and plant family as random effects to assess the extent to which phylogenetic relationships structure interaction probabilities, but this approach did not reveal strong family-level patterns. However, variability in the width of credible intervals across families may provide insight into the role of species-specific traits or ecological factors. For example, families with narrow credible intervals suggest more consistent interaction probabilities across their species, whereas families with wide intervals may contain

species with highly variable traits, such as differences in floral specialization, nesting behavior, or phenology, that influence their likelihood of interacting with particular partners. Note that the bee family Apidae includes both species with few (~ 25 plant species interactions; Appendix S1: Figure S1) and very high (> 100 plant species interactions), which also suggests that summarization at the family-level might not be as informative. This variation underscores the importance of considering species-level traits alongside broader phylogenetic patterns when interpreting interaction probabilities.

References

Brooks, S. P., and A. Gelman. 1998. General Methods for Monitoring Convergence of Iterative Simulations. Journal of Computational and Graphical Statistics 7:434–455.

de Valpine, P., C. Paciorek, D. Turek, N. Michaud, C. Anderson-Bergman, F. Obermeyer, C. W. Cortes, A. Rodrìguez, D. T. Lang, and S. Paganin. 2022. NIMBLE: MCMC, Particle Filtering, and Programmable Hierarchical Modeling.

de Valpine, P., D. Turek, C. J. Paciorek, C. Anderson-Bergman, D. T. Lang, and R. Bodik. 2017. Programming With Models: Writing Statistical Algorithms for General Model Structures With NIMBLE. Journal of Computational and Graphical Statistics 26:403–413.

DiRenzo, G. V., E. Hanks, and D. A. W. Miller. 2023. A practical guide to understanding and validating complex models using data simulations. Methods in Ecology and Evolution 14:203–217.

Falgout, J., Gordon, J., Lee, L., Williams, B., and USGS Advanced Research Computing. 2025. USGS Hovenweep supercomputer. DOI: https://doi.org/10.5066/P927BI7R.

R Core Team. 2023. R: A Language and Environment for Statistical Computing. R Foundation for Statistical Computing, Vienna, Austria.

Ruiz-Gutiérrez, V., E. F. Zipkin, and A. A. Dhondt. 2010. Occupancy dynamics in a tropical bird community: unexpectedly high forest use by birds classified as non-forest species. Journal of Applied Ecology 47:621–630.
