# Supplementary material for: Leveraging local species data, a global database, and an occupancy model to explore bee–plant interactions
Source: Ecol Appl. 2026 Mar 24;36(2):e70221. doi: 10.1002/eap.70221 (PMC13012871; doi:10.1002/eap.70221)
Supplement: Supplementary file 3 — Appendix S3. [file EAP-36-e70221-s003.pdf]

Appendix S3

Leveraging local species data, a global database, and an occupancy model to explore bee–plant interactions

Michelle J. Lee, Graziella V. DiRenzo, Chengyi Diao, Katja C. Seltmann

Ecological Applications

Figure S1 (next page). Networks visualizations for the bee family Apidae.

Any use of trade, firm, or product names is for descriptive purposes only and does not imply endorsement by the U.S. Government.

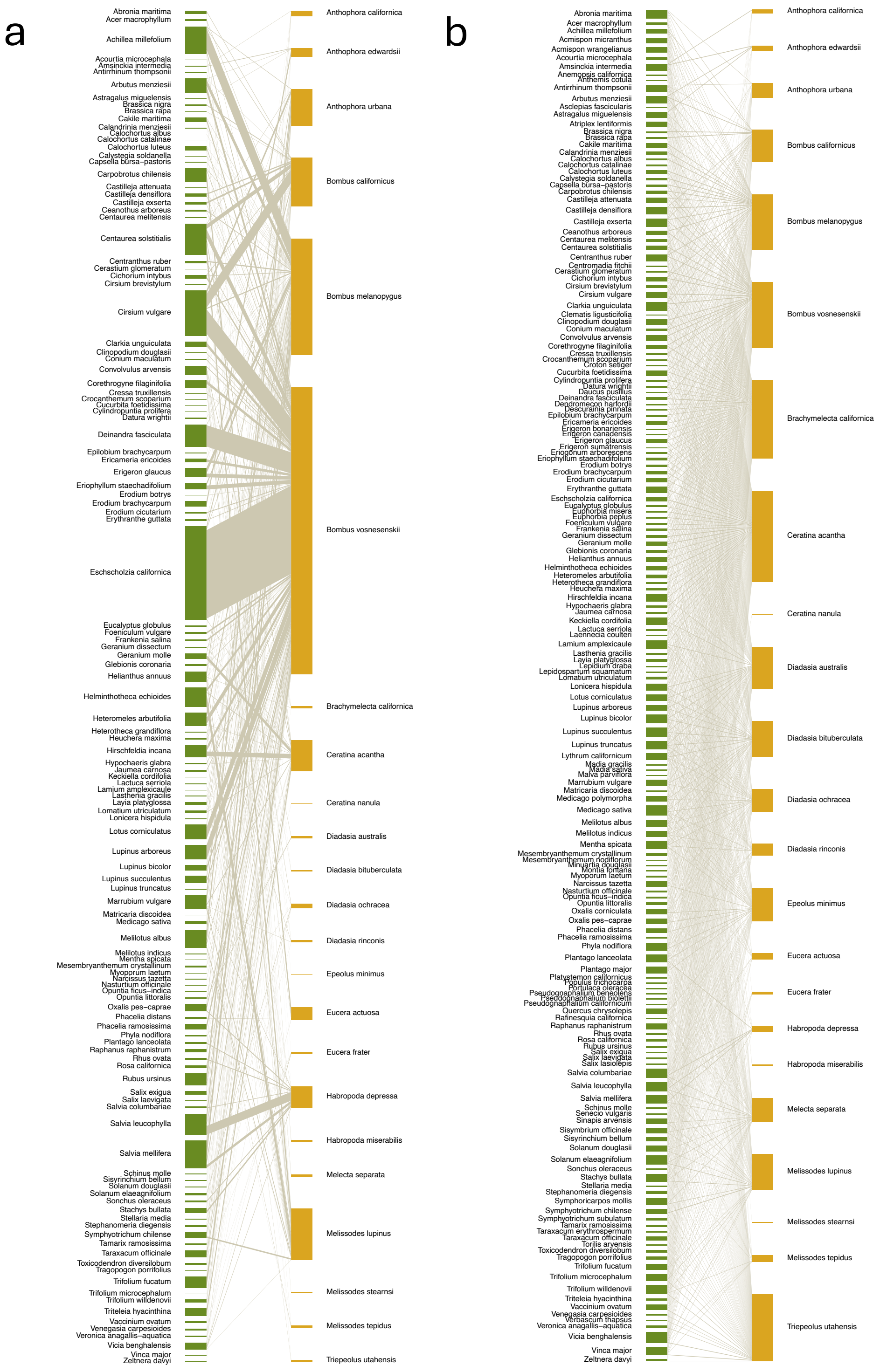
a
Abronia maritima
Acer macrophyllum
Achillea millefolium
Acourtia microcephala
Amsinckia intermedia
Antirrhinum thompsonii
Arbutus menziesii
Astragalus miguelensis
Brassica nigra
Brassica rapa
Cakile maritima
Calandrinia menziesii
Calochortus albus
Calochortus catalinae
Calochortus luteus
Calystegia soldanella
Capsella bursa−pastoris
Carpobrotus chilensis
Castilleja attenuata
Castilleja densiflora
Castilleja exserta
Ceanothus arboreus
Centaurea melitensis
Centaurea solstitialis
Centranthus ruber
Cerastium glomeratum
Cichorium intybus
Cirsium brevistylum
Cirsium vulgare
Clarkia unguiculata
Clinopodium douglasii
Conium maculatum
Convolvulus arvensis
Corethrogyne filaginifolia
Cressa truxillensis
Crocanthemum scoparium
Cucurbita foetidissima
Cylindropuntia prolifera
Datura wrightii
Deinandra fasciculata
Epilobium brachycarpum
Ericameria ericoides
Erigeron glaucus
Eriophyllum staechadifolium
Erodium botrys
Erodium brachycarpum
Erodium cicutarium
Erythranthe guttata
Eschscholzia californica
Eucalyptus globulus
Foeniculum vulgare
Frankenia salina
Geranium dissectum
Geranium molle
Glebionis coronaria
Helianthus annuus
Helminthotheca echioides
Heteromeles arbutifolia
Heterotheca grandiflora
Heuchera maxima
Hirschfeldia incana
Hypochaeris glabra
Jaumea carnosa
Keckiella cordifolia
Lactuca serriola
Lamium amplexicaule
Lasthenia gracilis
Layia platyglossa
Lomatium utriculatum
Lonicera hispidula
Lotus corniculatus
Lupinus arboreus
Lupinus bicolor
Lupinus succulentus
Lupinus truncatus
Marrubium vulgare
Matricaria discoidea
Medicago sativa
Melilotus albus
Melilotus indicus
Mentha spicata
Mesembryanthemum crystallinum
Myoporum laetum
Narcissus tazetta
Nasturtium officinale
Opuntia ficus−indica
Opuntia littoralis
Oxalis pes−caprae
Phacelia distans
Phacelia ramosissima
Phyla nodiflora
Plantago lanceolata
Raphanus raphanistrum
Rhus ovata
Rosa californica
Rubus ursinus
Salix exigua
Salix laevigata
Salvia columbariae
Salvia leucophylla
Salvia mellifera
Schinus molle
Sisyrinchium bellum
Solanum douglasii
Solanum elaeagnifolium
Sonchus oleraceus
Stachys bullata
Stellaria media
Stephanomeria diegensis
Symphyotrichum chilense
Tamarix ramosissima
Taraxacum officinale
Toxicodendron diversilobum
Tragopogon porrifolius
Trifolium fucatum
Trifolium microcephalum
Trifolium willdenovii
Triteleia hyacinthina
Vaccinium ovatum
Venegasia carpesioides
Veronica anagallis−aquatica
Vicia benghalensis
Vinca major
Zeltnera davyi
Anthophora californica
Anthophora edwardsii
Anthophora urbana
Bombus californicus
Bombus melanopygus
Bombus vosnesenskii
Brachymelecta californica
Ceratina acantha
Ceratina nanula
Diadasia australis
Diadasia bituberculata
Diadasia ochracea
Diadasia rinconis
Epeolus minimus
Eucera actuosa
Eucera frater
Habropoda depressa
Habropoda miserabilis
Melecta separata
Melissodes lupinus
Melissodes stearnsi
Melissodes tepidus
Triepeolus utahensis
b
Abronia maritima
Acer macrophyllum
Achillea millefolium
Acmispon micranthus
Acmispon wrangelianus
Acourtia microcephala
Amsinckia intermedia
Anemopsis californica
Anthemis cotula
Antirrhinum thompsonii
Arbutus menziesii
Asclepias fascicularis
Astragalus miguelensis
Atriplex lentiformis
Brassica nigra
Brassica rapa
Cakile maritima
Calandrinia menziesii
Calochortus albus
Calochortus catalinae
Calochortus luteus
Calystegia soldanella
Capsella bursa−pastoris
Carpobrotus chilensis
Castilleja attenuata
Castilleja densiflora
Castilleja exserta
Ceanothus arboreus
Centaurea melitensis
Centaurea solstitialis
Centranthus ruber
Centromadia fitchii
Cerastium glomeratum
Cichorium intybus
Cirsium brevistylum
Cirsium vulgare
Clarkia unguiculata
Clematis ligusticifolia
Clinopodium douglasii
Conium maculatum
Convolvulus arvensis
Corethrogyne filaginifolia
Cressa truxillensis
Crocanthemum scoparium
Croton setiger
Cucurbita foetidissima
Cylindropuntia prolifera
Datura wrightii
Daucus pusillus
Deinandra fasciculata
Dendromecon harfordii
Descurainia pinnata
Epilobium brachycarpum
Ericameria ericoides
Erigeron bonariensis
Erigeron canadensis
Erigeron glaucus
Erigeron sumatrensis
Eriogonum arborescens
Eriophyllum staechadifolium
Erodium botrys
Erodium brachycarpum
Erodium cicutarium
Erythranthe guttata
Eschscholzia californica
Eucalyptus globulus
Euphorbia misera
Euphorbia peplus
Foeniculum vulgare
Frankenia salina
Geranium dissectum
Geranium molle
Glebionis coronaria
Helianthus annuus
Helminthotheca echioides
Heteromeles arbutifolia
Heterotheca grandiflora
Heuchera maxima
Hirschfeldia incana
Hypochaeris glabra
Jaumea carnosa
Keckiella cordifolia
Lactuca serriola
Laennecia coulteri
Lamium amplexicaule
Lasthenia gracilis
Layia platyglossa
Lepidium draba
Lepidospartum squamatum
Lomatium utriculatum
Lonicera hispidula
Lotus corniculatus
Lupinus arboreus
Lupinus bicolor
Lupinus succulentus
Lupinus truncatus
Lythrum californicum
Madia gracilis
Madia sativa
Malva parviflora
Marrubium vulgare
Matricaria discoidea
Medicago polymorpha
Medicago sativa
Melilotus albus
Melilotus indicus
Mentha spicata
Mesembryanthemum crystallinum
Mesembryanthemum nodiflorum
Minuartia douglasii
Montia fontana
Myoporum laetum
Narcissus tazetta
Nasturtium officinale
Opuntia ficus−indica
Opuntia littoralis
Oxalis corniculata
Oxalis pes−caprae
Phacelia distans
Phacelia ramosissima
Phyla nodiflora
Plantago lanceolata
Plantago major
Platystemon californicus
Populus trichocarpa
Portulaca oleracea
Pseudognaphalium beneolens
Pseudognaphalium biolettii
Pseudognaphalium californicum
Quercus chrysolepis
Rafinesquia californica
Raphanus raphanistrum
Rhus ovata
Rosa californica
Rubus ursinus
Salix exigua
Salix laevigata
Salix lasiolepis
Salvia columbariae
Salvia leucophylla
Salvia mellifera
Schinus molle
Senecio vulgaris
Sinapis arvensis
Sisymbrium officinale
Sisyrinchium bellum
Solanum douglasii
Solanum elaeagnifolium
Sonchus oleraceus
Stachys bullata
Stellaria media
Stephanomeria diegensis
Symphoricarpos mollis
Symphyotrichum chilense
Symphyotrichum subulatum
Tamarix ramosissima
Taraxacum erythrospermum
Taraxacum officinale
Torilis arvensis
Toxicodendron diversilobum
Tragopogon porrifolius
Trifolium fucatum
Trifolium microcephalum
Trifolium willdenovii
Triteleia hyacinthina
Vaccinium ovatum
Venegasia carpesioides
Verbascum thapsus
Veronica anagallis−aquatica
Vicia benghalensis
Vinca major
Zeltnera davyi
Anthophora californica
Anthophora edwardsii
Anthophora urbana
Bombus californicus
Bombus melanopygus
Bombus vosnesenskii
Brachymelecta californica
Ceratina acantha
Ceratina nanula
Diadasia australis
Diadasia bituberculata
Diadasia ochracea
Diadasia rinconis
Epeolus minimus
Eucera actuosa
Eucera frater
Habropoda depressa
Habropoda miserabilis
Melecta separata
Melissodes lupinus
Melissodes stearnsi
Melissodes tepidus
Triepeolus utahensis
